# Scalable, Microwave‐Enabled Synthesis of Ternary W_x_Ti_1‐x_O_2_ and Heterostructured TiO_2_‐WO_3‐x_ Colloidal Nanocrystals: Carrier Dynamics and Photocatalytic Properties

**DOI:** 10.1002/advs.202514916

**Published:** 2025-12-16

**Authors:** Riccardo Scarfiello, Michele Guizzardi, Angela Fiore, Armando Genco, Concetta Nobile, Elisabetta Perrone, Sara Fernanda Orsini, Clémentine Fellah, Lucian Roiban, Marianna Bellardita, Anna Maria Venezia, Giulio Cerullo, Leonardo Palmisano, Luigi Carbone

**Affiliations:** ^1^ CNR NANOTEC – Institute of Nanotechnology c/o Campus Ecotekne University of Salento, via Monteroni Lecce 73100 Italy; ^2^ Center for Biomolecular Nanotechnologies Istituto Italiano di Tecnologia Arnesano (LE) 73010 Italy; ^3^ Centro Ricerche Brindisi Agenzia Nazionale per le nuove tecnologie l'energia e lo sviluppo economico sostenibile ENEA‐SSPT‐EC‐RMP c/o Cittadella della Ricerca S.S.7 "Appia" Km 706 Brindisi 72100 Italy; ^4^ ENS Lyon UMR 5276 LGL‐TPE Université Claude Bernard Lyon 1 CNRS Lyon 69626 France; ^5^ INSA Lyon Université Claude Bernard Lyon 1 CNRS MATEIS UMR5510 Villeurbanne France; ^6^ Dipartimento di Ingegneria Università di Palermo Viale delle Scienze ed. 6 Palermo 90128 Italy; ^7^ ISMN‐CNR Via Ugo la Malfa 153 Palermo 90146 Italy; ^8^ Dipartimento di Fisica Politecnico di Milano Piazza Leonardo da Vinci, 32 Milano 20133 Italy

**Keywords:** defective oxide heterostructures, hot carrier dynamics, microwave synthesis, photocatalysis, ternary oxide

## Abstract

In solar‐light‐driven chemical processes, designing ever‐new strategies for synthesizing functional, doped, or defective semiconducting oxide nanostructures remains key. Techniques like bandgap engineering and interface heterostructuring, among others, have driven the development of brand‐new synthetic schemes that enable efficient charge carrier extraction. In this context, microwave (MW) chemistry has effectively established bottom‐up synthetic strategies. This study details a fast MW hydroalcohothermal synthesis for the scalable production of ternary W_x_Ti_1‐x_O_2_ and heterostructured TiO_2_‐WO_3‐x_ colloidal nanocrystals. MWs allow for size control at the nanoscale, and, in the case of heterostructures, support the anisotropic nucleation of oxygen‐deficient plasmonic WO_3‐x_ nanobelts directly onto the surfaces of pre‐existing TiO_2_ seeds, establishing unique reaction pathways. Femtosecond transient absorption spectroscopy reveals the formation and ultrafast cooling of hot electrons within the plasmonic domains at near‐infrared (NIR) wavelengths. Both nanostructures exhibit significant photo‐oxidant activity toward 4‐methoxybenzyl alcohol in a liquid aerobic environment, concurrently demonstrating enhanced selectivity toward aldehyde products. While the ternary material shows activity and notable selectivity exclusively under UV excitation, the heterostructures provide compelling functionality, especially under solar‐simulated light irradiation. This superior performance is ascribed to the synergistic coupling of the NIR‐assisted photocatalytic effect driven by hot carriers, along with the photothermal effect arising from plasmon excitation.

## Graphical abstract

Two microwave‐assisted synthetic methods, specifically a one‐pot and a seeded‐growth approach, have been developed within a hydroalcoholic solution for the growth of single‐phase ternary W_x_Ti_1‐x_O_2_ and heterostructured TiO_2_‐WO_3‐x_ colloidal nanocrystals. Both types of nanocrystals show significant photocatalytic activity for 4‐methoxybenzyl alcohol oxidation and exhibit selectivity toward aldehyde products in a liquid aerobic environment, under UV and solar‐simulated light irradiation, respectively.

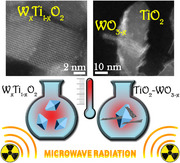

## 1 Introduction

Nanostructured semiconductors represent an excellent solution for a large variety of important solar‐light‐driven industrial processes and/or photo‐activated technologies, especially when involving earth‐abundant and non‐toxic materials.^[^ ^1^ ^]^ In this framework, TiO_2_ has for years proved to be one of the most strategic systems studied for a wide range of applications thanks to its reactivity, long‐term stability, and non‐toxicity.^[^ ^1^, ^2^, ^3^, ^4^, ^5^, ^6^ ^]^ However, the implementation of high‐performance light‐driven chemical processes is limited by some drawbacks such as the fast internal recombination of excitons and photo‐activity restricted to the UV range, which inhibit practical large‐scale applications. Several experimental solutions to improve the photo‐activity of nanostructured TiO_2_ have been proposed in the literature, including surface structuring,^[^ ^7^, ^8^ ^]^ crystal phase engineering,^[^ ^9^ ^]^ creation of defects,^[^ ^10^ ^]^ cation or anion doping,^[^ ^11^, ^12^, ^13^, ^14^ ^]^ and, last but not least, creation of heterojunctions.^[^ ^15^, ^16^, ^17^, ^18^, ^19^ ^]^

A more recent direction in the construction of nanocatalysts involves the synthesis of heterostructured plasmonic photocatalysts, in which a semiconductor is coupled with a plasmonic material, such as noble metal nanoparticles^[^ ^20^, ^21^, ^22^ ^]^ or self‐doped (or metal‐like) non‐metal oxides or sulfides^[^ ^23^, ^24^, ^25^ ^]^ that exhibit a localized surface plasmon resonance (LSPR). Hot carriers excited in the plasmonic domain can be injected into the semiconductor before their thermalization into a new equilibrium state,^[^ ^26^, ^27^ ^]^ and they are expected to play an important, though sometimes still unclear, role in catalytic reactions.^[^ ^28^ ^]^

In this scenario, tungsten is considered an ideal and versatile dopant/partner because it is soluble in large and variable amounts within the titania matrix, influencing the photochemistry and the photodegradation mechanism of TiO_2_ in different ways.^[^ ^29^, ^30^, ^31^, ^32^ ^]^ Thus, the inclusion of tungsten as a dopant, in low concentration,^[^ ^33^, ^34^, ^35^, ^36^, ^37^, ^38^ ^]^ or the heterostructuring of TiO_2_ with a secondary domain (such as tungsten trioxide or non‐stoichiometric phases)^[^ ^23^, ^39^, ^40^, ^41^, ^42^, ^43^, ^44^, ^45^, ^46^, ^47^, ^48^, ^49^ ^]^ represent resourceful alternatives to enhance photo‐activity. In particular, the latter allows the creation of type‐II heterojunctions between two semiconductors having staggered energy levels, which can facilitate the spatial separation and prolong the lifetime of photogenerated charge carriers at the interface, increasing the overall photochemical activity.^[^ ^19^ ^]^ For these reasons, TiO_2_‐WO_3‐x_ heterostructures as well as W‐doped TiO_2_ have received considerable attention for photoelectrochemical and photocatalytic properties.^[^ ^12^, ^13^, ^23^, ^31^, ^42^, ^50^, ^51^, ^52^, ^53^, ^54^, ^55^ ^]^ However, finely controlled synthesis of Ti‐ and W‐based nanocatalysts is not always straightforward. To date, most of the reported syntheses concern the fabrication of solid‐state W‐ and Ti‐based homo‐ and hetero‐structured nanocatalysts, where high temperature, pressure, and/or prolonged annealing conditions are often required to obtain high‐quality materials.^[^ ^12^, ^13^, ^23^, ^31^, ^44^, ^45^, ^46^, ^47^, ^48^, ^49^ ^]^ Microwave (MW)‐assisted syntheses offer major advantages over conventional solvothermal and hydrothermal methodologies, including drastically reduced processing time (down to minutes) and unique MW‐specific effects. These effects include: superheating of solvents, selective heating of specific reactants, exclusion of wall effects, and rapid‐heating kinetic events. These thermal side‐effects enable unconventional growth pathways and ensure the technique is scalable.^[^ ^56^ ^]^ These prerequisites are difficult to achieve with other synthetic routes. This technology can be vital for addressing a critical need: the absence of a scalable, economically feasible method for producing two‐phase‐segregated nano‐photocatalysts with fine size control and stable aqueous dispersibility, features that are necessary for broad commercial and biotechnological adoption.

We herein describe two robust synthetic routes, namely a one‐pot and a seeded‐growth approach, following a MW‐hydroalcohothermal procedure, capable of producing nanocrystals (NCs) of W_x_Ti_1‐x_O_2_ and TiO_2_‐WO_3‐x_ generated in aqueous media in an easily scalable manner, on a time scale of a few minutes. They expose insulating ligand‐free nanosized surfaces and exhibit selective photocatalytic performances in the UV and in the solar wavelength range, respectively. Depending on the synthetic strategy, we obtain either a non‐segregated, single‐phase W_x_Ti_1‐x_O_2_ ternary compound or a segregated, biphasic TiO_2_‐WO_3‐x_ nanoheterostructure, the latter featuring elongated belt‐like nanostructures of WO_3‐x_ nucleated onto (and/or folding) the original TiO_2_ seed. In TiO_2_‐WO_3‐x_, the nucleation of an oxygen‐deficient non‐stoichiometric anisotropic form of tungsten oxide extends the light absorption range in the near‐infrared (NIR) and exhibits a LSPR band, generating a degenerately doped plasmonic‐semiconducting nanoheterostructure. We use femtosecond pump‐probe spectroscopy, targeting both inter‐ and intraband transitions, demonstrating hot electron formation in the WO_3‐x_ domains and measuring electron‐electron scattering (≈30 fs) and electron‐phonon coupling (≈160 fs) timescales compatible with hot electron transfer and photothermal effects. Both types of nanostructures demonstrate significantly increased photo‐oxidant effectiveness for the partial oxidation of 4‐methoxybenzyl alcohol in liquid aerobic environments compared to commercial TiO_2_, and show improved selectivity toward aldehyde products. Nevertheless, while the ternary material is only active and selective under UV light, the heterostructured NCs offer significantly better performance, especially when exposed to solar‐simulated light irradiation. This makes our nanocrystalline heterostructures very promising for highly efficient and economically viable photocatalysis over a broad wavelength range, owing to the combined effects of MW‐enabled anisotropic growth, the NIR photocatalytic activity driven by hot carriers, and the photothermal plasmonic response.

## 2 Results and Discussion

### 2.1 Nanocrystals Synthesis and Structural and Chemical Characterization

The hydroalcohothermal preparation of W_x_Ti_1‐x_O_2_ NCs and TiO_2_‐WO_3‐x_ nanoarchitectures is addressed according to the two separate chloroalkoxide reaction paths described in the following. The first is a precursor co‐injection procedure, whereby titanium and tungsten precursors are admixed together in a three‐neck flask and then transferred into Teflon vessels for MW heating treatments; the second is a seeded‐growth approach consisting of the dropwise addition of a tungsten precursor solution to pre‐synthesized TiO_2_ NCs (unpurified TiO_2_ seeds), before MW heating. TiO_2_ seeds are prepared according to the procedure reported in Ref. [57] with some substantial changes, the most notable of which is achieved by operating with dielectric heating in a high‐loss tan δ hydroalcoholic solution. The usage of MW technology and airtight reactors allows operating at reaction temperatures well above the atmospheric‐pressure boiling point of each reagent, as well as running the in‐core heating of the vessel because of dielectric and conduction losses. The relatively high experimental temperatures and the consequent significant decrease of viscosities certainly reduce the MW‐induced contribution of the polar solvents (dielectric loss), whereas they enhance the input of the ionic species attributable to MWs (conduction loss). All the preparations are performed in a MW modular reactor, a system that allows scaling up a synthesis to several grams, performing many reactions at once.

**Figure 1:**
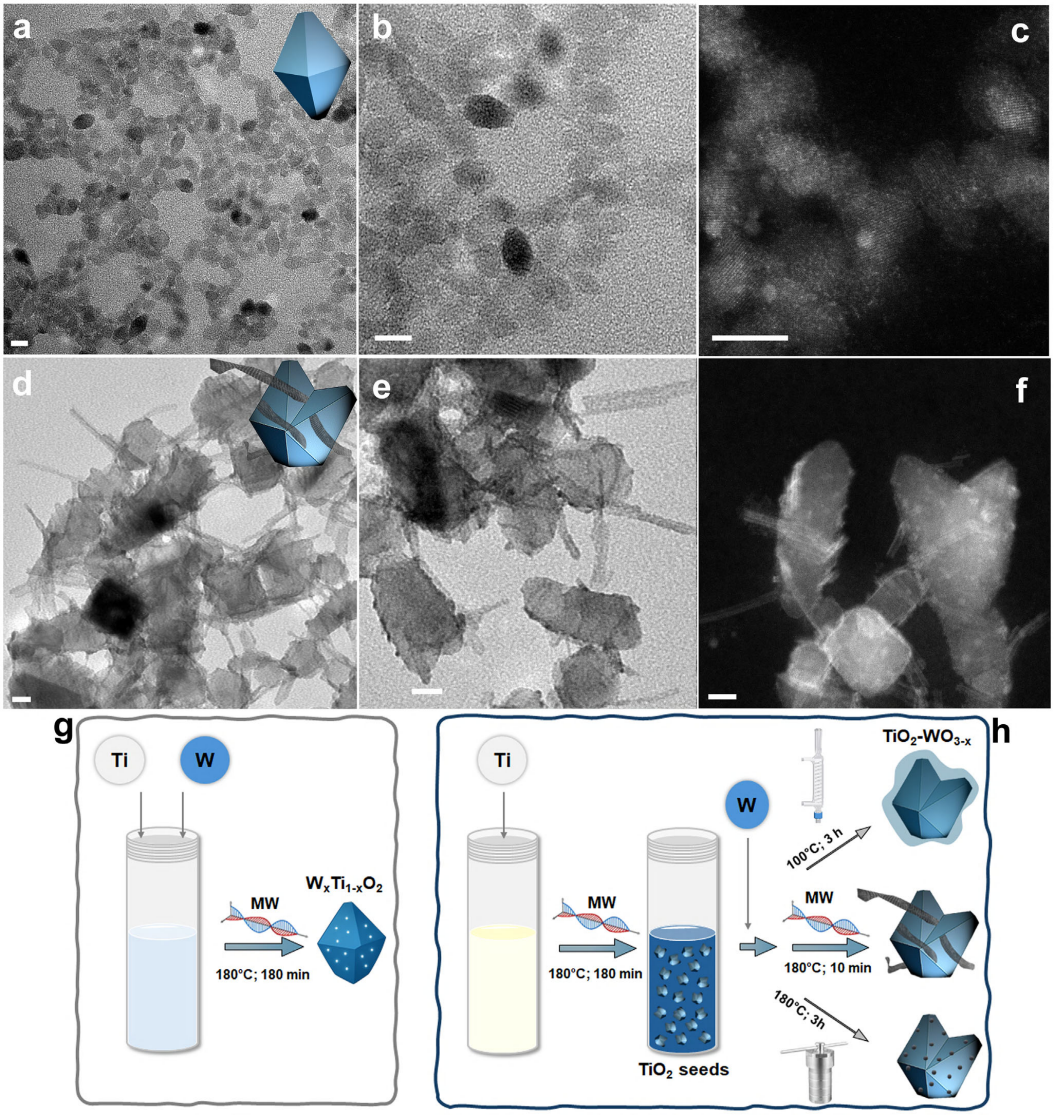
Low‐magnification electron micrographs of W_x_Ti_1‐x_O_2_ and TiO_2_‐WO_3‐x_ NCs. Conventional TEM (left and middle columns) and low‐magnification ADF‐STEM (right column) overviews of (a–c) ternary W_x_Ti_1‐x_O_2_ NCs obtained by one‐pot reaction between titanium (IV) isopropoxide and tungsten (VI) hexachloride. Bi‐pyramidal‐shaped NCs can be observed in the TEM pictures, whereas brighter spots due to W‐based domains are shown in the ADF‐STEM image. d–f) Heterostructured TiO_2_‐WO_3‐x_ sample, showing TiO_2_ seeds decorated with multiple WO_3‐x_ nanobelts, obtained through a seeded‐growth reaction between anatase TiO_2_ seeds and tungsten hexachloride solution. ADF‐STEM pictures featured a non‐homogeneous thin WO_3‐x_‐based shell surrounding the TiO_2_ surface and WO_3‐x_ nanobelts enveloping and/or protruding out of the TiO_2_ surface. Two sketches resembling sample morphologies are added to pictures (a,d). The scale bar is 10 nm. g,h) Reaction schemes illustrating the synthesis procedures of ternary W_x_Ti_1‐x_O_2_ and TiO_2_‐WO_3‐x_ NCs, respectively. Image (h) compares the growth methodology with other growth techniques, including autoclave‐based and in‐batch conventional heating. Further morphological details can be found in Figure S6 (Supporting Information).

The syntheses of W_x_Ti_1‐x_O_2_ NCs are developed via a characteristic hydroalcohothermal sol–gel approach where both metal precursors are pre‐mixed and slowly dropped into tetramethylammonium hydroxide (TMAH) water solution before MW heating. The mixing at 0°C of the TMAH and the Ti and W precursors promotes a slight grey clouding of the solution. Bipyramidal‐shaped ternary W_x_Ti_1‐x_O_2_ NCs in the anatase‐like crystal lattice are obtained (**Figure** **1**a–c; Figure S1a–c, Supporting Information). Low‐magnification annular dark‐field scanning transmission electron microscopy (ADF‐STEM) analysis performed on this sample reveals uniformity of distribution of tungsten heteroatoms over the entire volume of the NCs.

TiO_2_‐WO_3‐x_ nanodomain heterostructuring occurs via the seeded‐growth method by growing WO_3‐x_ nanobelts directly onto the TiO_2_ seeds. The original seeds are developed via a characteristic hydroalcohothermal sol–gel approach.^[^ ^57^ ^]^ The mixing at 0°C of the TMAH and the titanium solutions promotes a slight white clouding; then, the dispersion is transferred for MW processing (Figure S1d–f, Supporting Information). Afterward, an alcoholic solution of WCl_6_ is injected into a volume of non‐purified TiO_2_ NCs (see the Experimental Section for details), and then exposed to MWs again. The as‐developed samples show heterostructured domains. Specifically, pristine anatase TiO_2_ seeds are surrounded by W‐atom‐based small domains randomly located on their surface, as well as by WO_3‐x_ nanobelts as enveloping and/or protruding out of the surface of TiO_2_ nanostructures (Figure 1d–f; Figure S1g–i, Supporting Information). Upon increasing the amount of injected tungsten, the extent of heterostructuring increases, as evidenced by the larger build‐up of WO_3‐x_ nanobelts onto the titania surface (Figure S2, Supporting Information). Similarly, a less intricate network made by several hundred nanometers long belt‐like strands of tungsten oxide is formed when the concentrations of all the reagents are accordingly increased (Figures S3 and S4, Supporting Information). The latter is explained by a mass effect of the highly reactive WCl_6_ under the action of MW heating. Interestingly, no WO_3‐x_ nanodomains form when the synthesis is performed employing purified (three steps of filtration through 50 kDa MWCO) and water‐dispersed TiO_2_ seeds; indeed, the injection of an isopropanol solution of tungsten precursor does not promote the formation of WO_3‐x_ nanobelts and the formation of heterostructures (Figure S5, Supporting Information). This is a clear indication that the extra TMAH base, residual on the TiO_2_ seed's surface, is necessary and activated by MW to promote the heterogeneous nucleation of WO_3‐x_ NCs. Only at high concentrations of W precursor, very few nanobelts are visible (Figure S5, Supporting Information). WO_3‐x_ NCs develop in solution also under conditions of a lack of seeds formerly injected, as already reported elsewhere.^[^ ^58^ ^]^

The effectiveness of MW, mostly in the case of heterostructures, is validated upon performing two specific sets of experiments. First, the conventional convective heating method (using a characteristic heating mantle, with the only limitation of the operative reaction temperature of T = 100°C for 3 h) is compared to MW irradiation at 100°C for 3 h in airtight reactors tested with the same synthetic mixture. Samples generated with the conventional reflux method do not show any WO_3‐x_ nanobelt; only an irregularly thick shell encapsulating the TiO_2_ seeds and interconnecting them is identified (Figure S6a, Supporting Information). On the other hand, treating the same mixture at 100°C by MW irradiation promotes the decoration of TiO_2_ with WO_3‐x_ nanobelts. The generated belts turned out to be shorter and denser, but undoubtedly reveal their characteristic 1D nature (Figure S6b, Supporting Information). Furthermore, a sequential control experiment has been run. The synthetic mixture has been first refluxed (100°C, 3 h, 1 atm) and then MW irradiated (180°C, 10 min). TiO_2_ seeds surrounded by a W‐based shell are identified as intermediates between the two steps, which evolve into TiO_2_‐WO_3‐x_ nanoheterostructures under MW action (Figure S6c, Supporting Information). These results establish the parallel effective action carried on by MWs and temperature. Similar approaches have been performed in an autoclave as well; syntheses have been achieved at 180°C for 30 min, 3 h, and 24 h. In all cases, the deposition of spherical dots of tungsten oxide on the surface of the seeds has been observed regardless of the time of growth, without any example of 1D linear growth (Figure S6d–f, Supporting Information). According to this procedure, a large density of dots free in solution is equally evident, particularly in the early stages of growth (Figure S6d, Supporting Information). This corroborates the thesis that the high temperature is the dominating force driving the nucleation of the new phase, whereas MW uniquely contributes to both selecting the seed surface as the preferential place for growth and delivering high reactivity (and its large availability, thereby) to the tungsten precursor to promote a kinetic path to the growth. Even a significant amount of WCl_6_ (Figure S6g–i, Supporting Information), keeping constant the concentration of all the other reagents, does not promote the anisotropic development of WO_3‐x_ domains in the autoclave apparatus. A NC growth mechanism is suggested in the Supporting Information.

**Figure 2:**
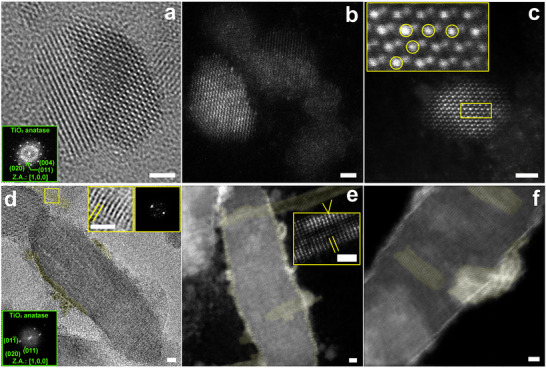
HRTEM (left column) and ADF‐STEM (middle and right columns) of two different Ti‐ and W‐based oxide nanostructures. a–c) Ternary W_x_Ti_1‐x_O_2_ sample. (d‐f) TiO_2_‐WO_3‐x_ heterostructured NCs composed of an anatase TiO_2_ crystal seed, surrounded by a WO_3‐x_ thin discontinuous shell, from which WO_3‐x_ belt‐like nanostructures, with a monoclinic crystalline arrangement, are observed to come out. The scale bar is 2 nm.

High‐resolution transmission electron microscopy (HRTEM) and ADF‐STEM investigations were carried out on our samples to provide structural and compositional information about colloidal nanostructures. A combination of both analysis techniques allows unambiguous indications about the hybrid nature of our Ti‐ and W‐based oxide nanoarchitectures and their crystalline morphology, as reported in **Figure** **2**. Figure 2a reports a representative bipyramidal‐shaped ternary W_x_Ti_1‐x_O_2_ NC. The HRTEM analysis shows that the nanostructure has a crystal arrangement embedded into a TiO_2_ anatase‐like crystal lattice as reported in the diffraction pattern in the inset, where characteristic anatase crystallographic planes are highlighted. Further, ADF‐STEM investigations show examples of NCs with a TiO_2_ anatase crystal geometry where atomic columns containing W‐inclusions are evident. Figure 2b,c shows an anatase crystal structure, where some atomic columns present a stronger intensity profile with respect to the surrounding others (see yellow circles in Figure 2c). Such changes in the image pattern are ascribed to the presence (likely in place of Ti) of W atoms, allowing the three different species (Ti, W, O) to be organized in an anatase crystal lattice. This is not unlikely, given the proper matching of the ionic radius of W^+6^ and Ti^+4^, which, therefore, does not entail a sensible change of crystalline habit. Based on such HRTEM and ADF‐STEM studies, then confirmed by chemical elemental analysis (see below), we deduce the ternary W_x_Ti_1‐x_O_2_ nature of the sample. HRTEM investigations of heterostructured samples (Figure 2d) confirm that the anatase crystalline nature of the starting TiO_2_ NC seeds is preserved in the final product. Very small domains (see yellow lines/shadows in Figure 2d) onto the TiO_2_ NC edges and thin 2D belt‐shaped structures protruding out of the seed surface can be noticed in the HRTEM image (zoom inset in Figure 2d,e). Advanced STEM investigations (Figure 2e,f) provide a direct observation and identification of such domains. Based on Z‐contrast considerations, non‐crystalline W atomic domains and monoclinic WO_3‐x_ nanobelt‐shaped structures along the whole TiO_2_ seed surface (yellow shadows for WO_3‐x_ nanobelts in Figure 2e,f) can be observed. The image intensity pattern shows both W‐based atomic domains, thickening on the TiO_2_ seed edges and forming a thin, irregular, and discontinuous‐in‐thickness shell, and WO_3‐x_ nanobelts, either enveloping or protruding out of the TiO_2_ crystal surface. The differently thick shell surrounding the TiO_2_ seed is expected to play a crucial role by providing suitable nucleation sites for the WO_3‐x_ nanobelts, which typically have a width of a few nanometers (≈2÷4 nm) and variable length, ranging from some nanometers to a few tens of nanometers. Besides, they are thin as compared to the thicker and larger original TiO_2_ seeds and feature a sort of flexibility allowing, in some cases, their wrapping around the TiO_2_ surface (Figure 2f). Additional low‐magnification high‐resolution ADF‐STEM overviews of the TiO_2_‐WO_3‐x_ heterostructured samples are reported in Figure S7 (Supporting Information) for a more exhaustive summary. Details about the non‐uniform shell surrounding the seeds are shown in Figure S8 (see orange arrows, Supporting Information); areas of the layer where the thickness is significantly denser appear as yellow shadows (Figure 2d–f; Figure S8, Supporting Information).

Images in Figure S9 (Supporting Information) correlate structural and chemical information of synthesized NCs. The powder X‐ray diffraction (XRD) patterns (Figure S9a, Supporting Information) of W_x_Ti_1‐x_O_2_ show reflections related to TiO_2_ anatase consistent with W‐ions substitution in lieu of titanium with a limited extent of doping (see Table S1, Supporting Information for quantitative inductively coupled plasma‐atomic emission spectroscopy (ICP‐AES) analysis; W_x_Ti_1‐x_O_2_ and TiO_2_‐WO_3‐x_ have revealed a tungsten molar content of 2.5 % and 39 %, respectively).^[^ ^59^ ^]^ TiO_2_‐WO_3‐x_ nanoheterostructures show two weak reflections (Bragg angle 2θ = 23.1°, 27.9°, specifically) ascribed to the monoclinic WO_3_ phase.^[^ ^44^, ^60^, ^61^, ^62^ ^]^ Figure S9b (Supporting Information) reports the X‐ray photoelectron spectroscopy (XPS) spectra of W_x_Ti_1‐x_O_2_ and TiO_2_‐WO_3‐x_ NCs; all signals are summarized in Table S2 (Supporting Information). Quantitative analyses in TiO_2_‐WO_3‐x_ provide 9 % of W^5+^. Differently, the W 4f spectrum of W_x_Ti_1‐x_O_2_ exhibits only one rather weak and broad doublet with W 4f_7/2_ binding energy at 36.7 eV, ascribed to only W^6+^. A residual amount of nitrogen is observed in the case of TiO_2_ seeds, possibly derived from the synthetic path. Raman spectra are reported in Figure S9c–e (Supporting Information). Characteristic peaks of the anatase phase are recorded in pristine TiO_2_ seeds.^[^ ^63^ ^]^ In the presence of tungsten, a slight shift of the TiO_2_ main peak at 144.5 cm^−1^ (1‐Eg) toward higher wavenumbers with respect to bare TiO_2_ seeds is observed. The shift is attributed to the presence of oxygen vacancies in the crystal lattice as a consequence of W‐ions inclusion, replacing Ti‐ions, into the TiO_2_ crystal lattice^[^ ^12^ ^]^ and to the subsequent expansion of the anatase unit cell.^[^ ^64^, ^65^ ^]^ Also, a broadening of the anatase band (144.5 cm^−1^) is shown in all experimental cases. In the case of heterostructures, the peak at ≈ 806 cm^−1^ is the clearest signal attributed to the monoclinic WO_3‐x_.^[^ ^50^, ^66^, ^67^, ^68^ ^]^ The other band of the tungsten oxide phase at 714 cm^−1^ is instead covered by that of anatase. More details can be found in the section on structural and chemical characterization in the Supporting Information.

### 2.2 Optical Characterization: Steady‐State and Time‐Resolved Spectroscopic Analyses

**Figure 3:**
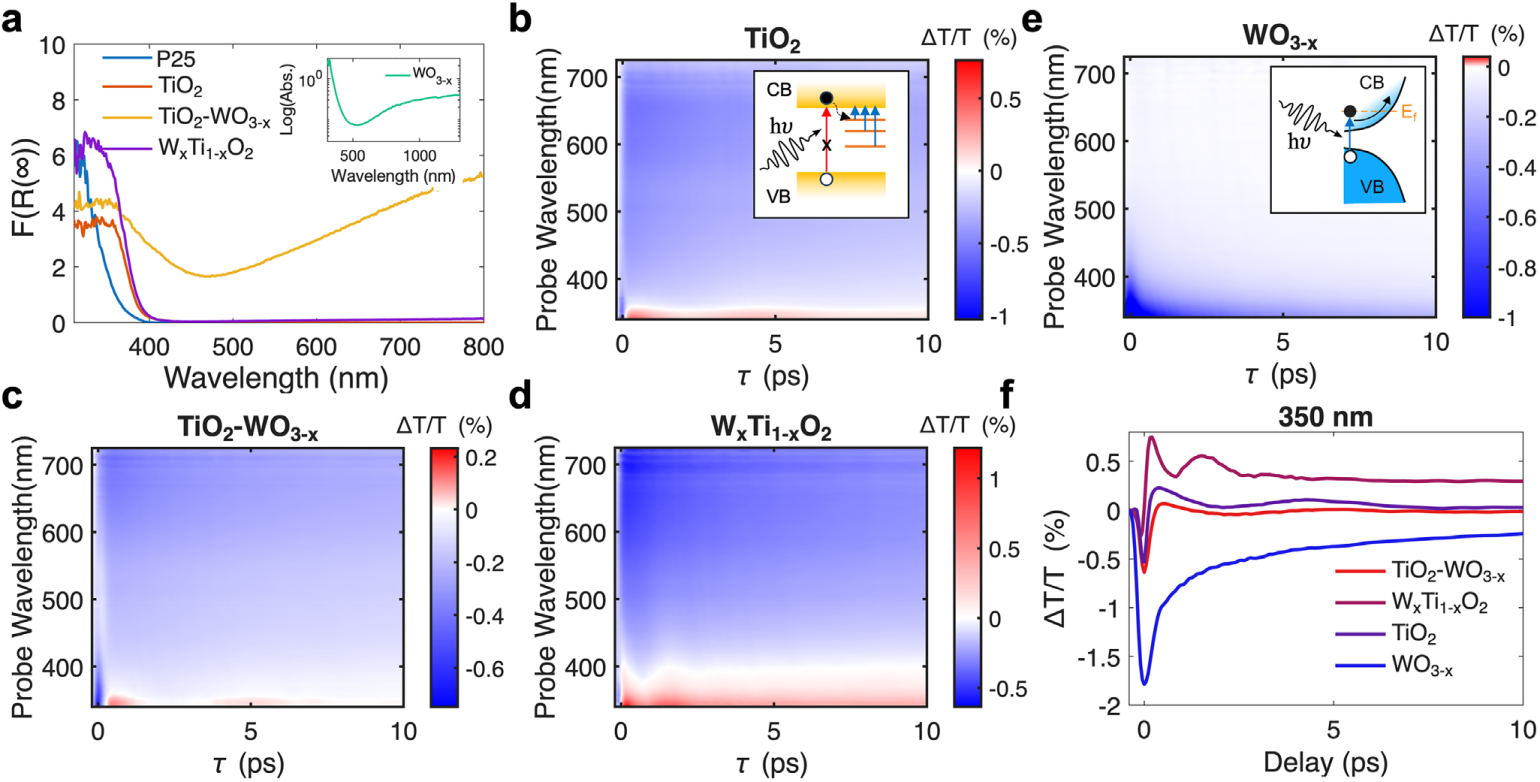
a) Absorbance curves of the samples derived from the thin film reflectance measurements through the Kubelka–Munk treatment. F(R_∞_) is the Kubelka–Munk function, whereby F(R_∞_)=K/S=absorption coefficient/scattering coefficient; typically, it reflects the true absorption spectra. The inset shows the vis‐NIR absorption spectrum of WO_3‐x_ in solution. a–e) ΔT/T maps of (b) TiO_2_, (c) WO_3‐x_, (d) heterostructure of TiO_2_‐WO_3‐x_, and (e) W_x_Ti_1‐x_O_2_ ternary compound, excited at 266 nm. The insets of (b,c) show schematics of the out‐of‐equilibrium absorption processes in TiO_2_ and WO_3‐x_. f) Temporal dynamics extracted by fixing the probe wavelength at 350 nm for every sample.

The optical properties of the as‐developed nanostructures are studied through UV–vis Diffuse Reflectance (DR) spectroscopy of dry powders (Figure S10a, Supporting Information) and absorbance spectroscopy of samples dispersed in liquid media (Figure S10c,d, Supporting Information). In liquid media, especially when colloidal nanomaterials are well‐dispersed (as in our case), particles are suspended individually, which minimizes light scattering. In contrast, in dry powder samples, particles tend to aggregate, and the lack of a solvent leads to increased light scattering due to larger clusters and surface roughness. This scattering can distort or broaden the measured absorption features. Additionally, the solvent or surface coordination can affect the electronic environment of the nanomaterials, potentially shifting absorption bands or altering their intensity. The reflectance edge of the ternary W_x_Ti_1‐x_O_2_, as well as that of the original TiO_2_ seeds, redshifts compared to commercial TiO_2_, because of intraband gap states resulting from tungsten doping (inclusion) and/or oxygen vacancies. A more significant shift is observed for TiO_2_‐WO_3‐x_ heterostructures due to the presence of the WO_3‐x_ nanobelts on the TiO_2_ surface.^[^ ^16^, ^32^, ^69^, ^70^ ^]^ Clear evidence of vis‐NIR (above 460 nm) active LSPR of TiO_2_‐WO_3‐x_ is observed, especially in dry powder (**Figure** **3**a; Figure S10a, Supporting Information) and in water‐dispersed heterostructures enriched in W content (Figure S10d–h, Supporting Information). The Tauc plot method by applying the Kubelka–Munk function to the DR spectra (Figure S10b, Supporting Information) is adopted to estimate the E_g_ bandgap energies.^[^ ^50^ ^]^ The derived E_g_ values are reported in the last column of Table S1 (Supporting Information). The TiO_2_ seed shows a reduced energy bandgap compared to the commercial P25 TiO_2_ photocatalyst, 3.08 eV versus 3.18 eV, respectively. The presence of tungsten in the structure has a different effect on the energy bandgap. When it forms a ternary nanostructure, negligible influences on the energy bandgap can be measured (3.05 eV), while a consistent red‐shifting to 2.69 eV is evident from reflectance measurements of heterostructures. In the latter case, instead of accounting for the intersection between the linear fit and the photon energy axis in the Kubelka–Munk treatment, the baseline approach, as reported by Macyk et al.,^[^ ^71^ ^]^ has been considered because it is more pertinent for systems that show considerable absorbance at an energy below Eg, as expected because of the presence of WO_3‐x_ (Figures S10b and S11, Supporting Information). Further details can be found in the section on optical characterization in the Supporting Information.

To unveil the dynamics of charge carriers, we performed ultrafast pump‐probe spectroscopy experiments on samples dispersed in aqueous solutions. We study the electronic landscape of the materials after optical excitation with energy‐tunable femtosecond pump pulses while probing the differential transmission (ΔT/T) of the samples using broadband visible pulses (more details about the technique can be found in the Experimental Section). Figure 3b–f shows the transient ΔT/T maps measured while exciting the different samples at 266 nm (4.66 eV). With such a high photon energy, well above the absorption onsets of both TiO_2_ and WO_3‐x_, the dynamics of interband transitions for the different compounds can be investigated. Figure 3b shows the ΔT/T map of bare TiO_2_. The positive ΔT/T signal observed below 350 nm immediately after the zero time delay is ascribed to a photo‐bleaching of the direct transitions above the bandgap due to Pauli blocking, while the broad negative band observed at longer wavelengths is assigned to photo‐induced absorption from defect states (see inset in Figure 3b).^[^ ^72^ ^]^ Figure 3e displays the results of the pump‐probe experiment performed on the bare WO_3‐x_ (see inset of Figure 3a for the static absorption of WO_3‐x_), which differently shows a strong negative signal at short wavelengths and at short time delays, due to the so‐called inverse Moss–Burstein effect, which quickly decays on the sub‐picosecond timescale.^[^ ^24^, ^73^ ^]^ In this naturally doped (degenerate) semiconductor, the Fermi level lies in the conduction band (CB).^[^ ^74^ ^]^ In oxygen‐deficient WO_3‐x_, oxygen vacancies introduce free electrons that populate the CB. Femtosecond spectroscopy^[^ ^75^ ^]^ combined with DFT calculations^[^ ^76^ ^]^ directly demonstrates that the Fermi level shifts into the tungsten‐based CB when oxygen vacancies are introduced, transforming the material from a wide‐gap semiconductor to a metallic conductor. When the pump pulses hit the sample, electrons from the valence band (VB) are promoted to the CB, creating an out‐of‐equilibrium electron distribution. The scattering of the newly excited electrons with those already present in the CB generates a hot Fermi‐Dirac distribution, emptying some states at the bottom of the CB, hence allowing photo‐induced direct transitions from the VB, resulting in the negative signals at short wavelengths in our pump‐probe measurements. The progressive blueshift of the signal upon increasing the time delay is related to the relaxation of the hot electrons’ distribution in the CB. This result evidences the formation of hot carriers in the WO_3‐x_ (see inset in Figure 3e), confirming the plasmonic response measured in static absorption induced by oxygen vacancies (see inset in Figure 3a). Figure 3c shows the pump‐probe map taken on the TiO_2_‐WO_3‐x_ heterostructure, which displays a similar response compared to the bare TiO_2_. On the other hand, the ultrafast dynamics of the ternary compound shown in Figure 3d differ significantly from the latter: the absence of a strong negative signal at short wavelengths indicates that the Moss–Burstein effect seen in WO_3‐x_ is not present, confirming the formation of the ternary compound instead of the WO_3‐x_ species, and fast coherent oscillations appear at time delays shorter than 5 ps, as evident in the isosbestic white line in the map of Figure 3d.

Comparing the temporal dynamics up to 10 ps, taken at 350 nm (Figure 3f), we observe oscillations in the traces of TiO_2_, TiO_2_‐WO_3‐x,_ and W_x_Ti_1‐x_O_2_, which are related to impulsively excited coherent acoustic phonons in the materials.^[^ ^77^ ^]^ In semiconductors, the coupling of the electronic band structure to lattice vibrations via coherent phonons gives rise to strong oscillatory modifications of the dielectric constant of the material. Using pump‐probe spectroscopy, coherent phonons can be photoexcited by ultrashort light pulses, producing oscillations in the transient reflectivity or transmissivity signals, thus revealing fundamental insight into electron‐phonon interactions and structural properties of the material.^[^ ^78^, ^79^ ^]^ Coherent oscillations are not visible in the pump‐probe traces of WO_3‐x_, probably because of the small lateral size of the nanobelts, leading to an overdamped phononic response.^[^ ^80^ ^]^ The oscillation period in the TiO_2_ and the heterostructure is substantially the same, but the ternary compound shows a much shorter period. In particular, while the low‐frequency (period: ≈4 ps, frequency: ≈0.25 THz or 8.3 cm^−1^) damped oscillation that we observed in TiO_2_ is very similar to previous reports using NCs with comparable size,^[^ ^81^ ^]^ the oscillation frequency is more than doubled in W_x_Ti_1‐x_O_2_ (period: ≈1.8 ps, frequency: ≈0.55 THz or 18.3 cm^−1^). Understanding the photoexcited coherent phonon behavior in NCs is currently an open challenge due to the number of different parameters influencing it,^[^ ^82^ ^]^ but we suggest that in this case it may be attributed to W‐ions inclusion (Ti‐ions replacement, to be more precise) into TiO_2_ anatase crystal lattice,^[^ ^12^ ^]^ which produces a decrease of the bond length, increasing the stiffness of the material and thus the speed of sound and the acoustic phonon frequency.^[^ ^83^ ^]^ Another possible explanation is related to the smaller size of crystallites in ternary NCs compared to the TiO_2_, as a consequence of W‐inclusion, which can lead to higher acoustic phonon frequencies.^[^ ^80^ ^]^ It is worth underlining that these low‐frequency phonons could not be detected in our Raman measurements shown in Figure S9c–e (Supporting Information) since they are too close to the laser line to be resolved by our setup.

**Figure 4:**
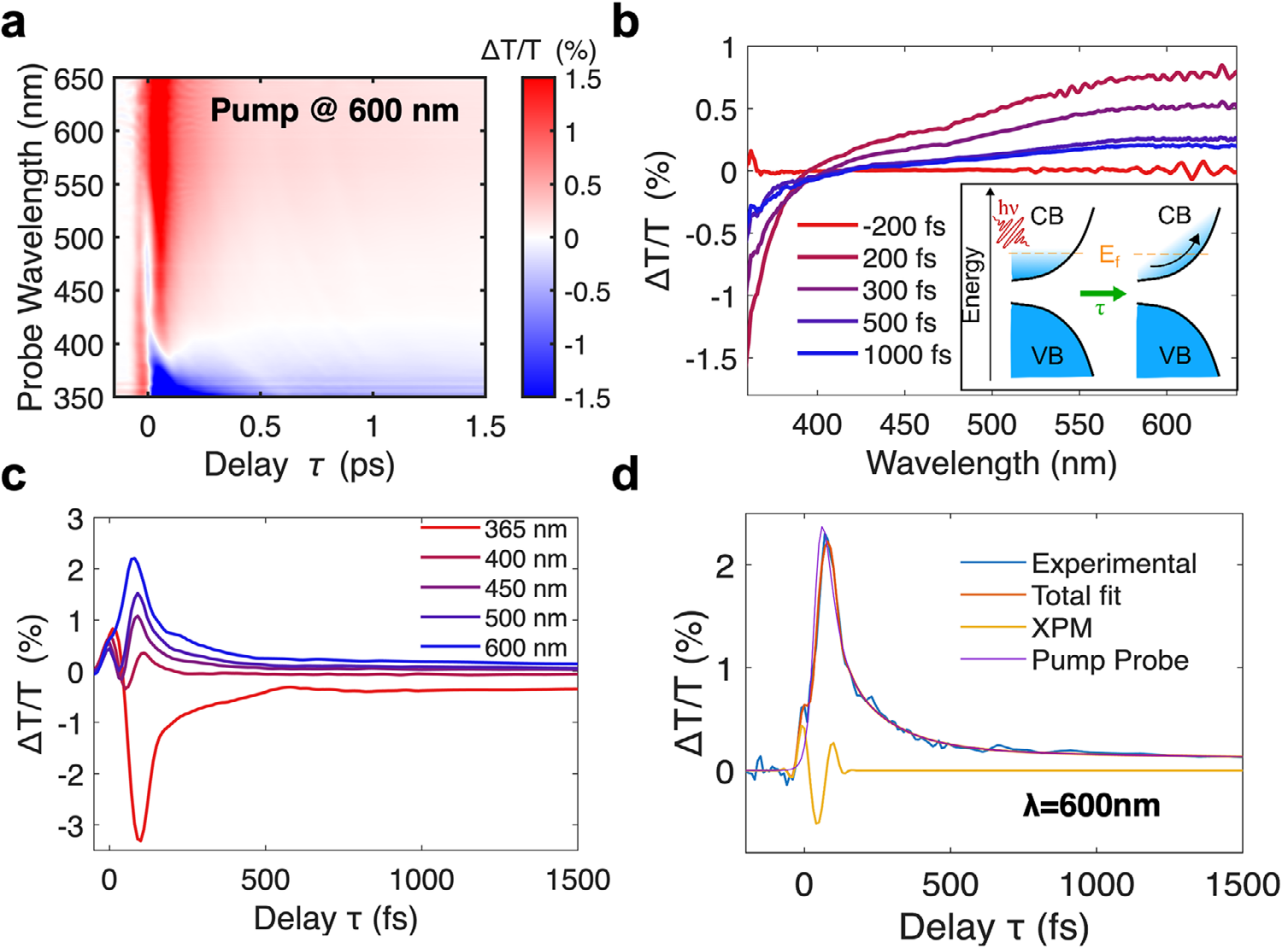
a) Pump‐probe transient transmission map exciting the plasmon resonance of the TiO_2_‐WO_3‐x_ heterostructure with visible pulses tuned at 600 nm, compressed to < 20 fs. b) Spectral cuts of the same map at different delay times. Inset: a sketch of the Moss–Burstein effect. c) Relaxation dynamics at different wavelengths. d) Fit of the trace at 600 nm showing the artifact removal.

After discussing the ultrafast response of the samples under interband excitation, we focus on the transient reflectivity experiments performed on the TiO_2_‐WO_3‐x_ heterostructure by pumping directly the plasmonic resonance of the WO_3‐x_ domains at 600 nm (below the energy bandgap of both materials). To observe the ultrafast sub‐100 fs dynamics characteristic of plasmonic systems, we used ultrashort pump pulses (pulse width < 20 fs), produced by an optical parametric amplifier (see Experimental Section for details). **Figure** **4**a presents the ΔT/T map of the heterostructure resulting from the plasmon excitation with visible pulses. The plasmonic response results in a different dynamic behavior compared with interband excitation. At approximately zero delay, we observe a strong cross‐phase modulation (XPM) artifact that can be removed in post‐processing, as discussed in the Experimental Section. More interestingly, at slightly longer times, a clear signal directly related to ultrafast plasmonic dynamics appears, which is nearly extinguished after 1 ps. Figure 4b plots the spectral evolution of the system, showing a positive ΔT/T signal changing sign at ≈400 nm, which we ascribe to a bleaching and a broadening of the plasmon resonance upon intraband excitation, due to the ultrafast excitation of a hot electron distribution in the plasmonic material (see inset in Figure 3b).^[^ ^84^ ^]^ We measured a similar behavior in the bare WO_3‐x_ samples (Figure S12, Supporting Information), confirming that such ultrafast nonlinear response is attributed to the plasmonic domain in the heterostructure. Such a signal was completely absent in the bare TiO_2_ or the ternary compound lacking plasmonic resonance (Figure S12, Supporting Information).

As discussed above, the WO_3‐x_ has a large electron density in the CB characteristic of degenerate semiconductors, leading to a broad plasmonic absorption resonance in the heterostructure that goes from the visible to the NIR (Figure 3a; Figure S10a,d, Supporting Information), well below the interband transition. When the pump pulse is absorbed by the sample, the excited plasmon creates a charge wave that propagates coherently inside the nanomaterial. On the ≈20 fs timescale, the plasmons dephase, generating an out‐of‐equilibrium distribution of hot electrons^[^ ^85^ ^]^ that rapidly thermalize in a hot Fermi‐Dirac distribution, on a time scale of ≈100 fs.^[^ ^86^ ^]^ Afterward, the hot carriers relax through electron‐phonon scattering, releasing the excess energy. The latter process typically takes place on a time scale of a few hundred fs, as also seen in this material (Figure 4c,d).^[^ ^87^ ^]^ At even longer time scales (> 1 ps), the lattice cools down due to phonon‐phonon scattering. Excited carriers can be generated at different energies by plasmon dephasing; while the so‐called Drude electrons are close to the Fermi level, the actual hot electrons are promoted up to an energy equal to that of the incoming photons above the Fermi level. In the latter case, charge carriers are generated at much higher energy compared to the ones possibly formed by an interband excitation, being more reactive and easily transferable to coupled materials,^[^ ^22^ ^]^ overcoming the Schottky barriers (indirect transfer), or injected into electronic states of molecules surface‐adsorbed onto the metallic domain (direct transfer, chemical interface damping). Since the VB is completely filled in WO_3‐x_, hot holes would then be available in the CB of the WO_3‐x_ domain for oxidation reactions. Energetic carriers are generated very close to the surface of the WO_3‐x_ domain; ultrathin (2–3 nm) slabs of WO_3‐x_ (Figures S7 and S8, Supporting Information) can operate as very efficient electron injectors (and nanoantennas as well) from the metal nanostructure into the semiconductor and/or surface molecule, as previously demonstrated.^[^ ^88^ ^]^ In our case, the aforementioned effects of the plasmonic excitation are key to enhancing the photocatalytic properties of the heterostructure (see below).

In Figure 4c, we report the relaxation dynamics for the heterostructure measured at different wavelengths, which show a multiexponential decay, as expected. In Figure 4d, we show an example of ΔT/T dynamics retrieval from the total temporal trace, taken in this case at a probe wavelength of 600 nm. Owing to our ultrashort pulse duration, we measured a first exponential decay with an ultrafast time constant of ≈30 fs, which we ascribe to electron‐electron scattering processes. In agreement with the literature, we also observe a second exponential decay related to electron‐phonon scattering, featuring a longer time constant of ≈160 fs.^[^ ^89^ ^]^ We also performed the same experiments by exciting the NIR part of the plasmonic resonance at 1000 nm in four types of heterostructures with different Ti‐to‐W content ratios (Figure S13, Supporting Information). We see a similar response for infrared excitation compared to the visible one, confirming the broad operational bandwidth of the plasmonic effects in the NIR, with increased signal and a slight spectral tuning of the plasmonic behavior increasing the W content, as also pointed out by the steady‐state absorbance spectra in Figure S10d (Supporting Information). Exciting the plasmon in the infrared makes the generation of hot electrons from Landau damping easier, and so could boost electron transfer.^[^ ^90^ ^]^ The exceptional tunability of the plasmon resonance and the formation of hot charge carriers point out the potential of this plasmonic (degenerate semiconductor)‐semiconductor nanocrystalline heterostructures, offering a new degree of freedom for infrared photocatalysis and plasmonic light‐harvesting.

### 2.3 Photo‐Oxidant Activity of Synthesized NCs in Aqueous Solution

**Figure 5:**
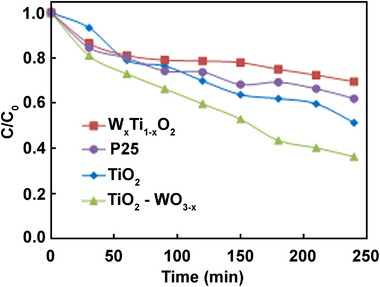
Conversion of 4‐MBA over irradiation time under simulated solar light irradiation in the presence of the different photocatalysts.

**Tables** **1** and S3 (Supporting Information) report the liquid‐phase experimental results from the heterogeneous photocatalytic partial oxidation of the 4‐MBA probe under simulated solar and pure UV irradiation obtained with W_x_Ti_1‐x_O_2_ and TiO_2_‐WO_3‐x_ NCs, respectively; these results are compared with the photo‐oxidation performances of original TiO_2_ seeds and commercial bare P25. The extent of the alcohol conversion after 4 h of irradiation is assessed (Table 1, X_t = 4 h_). Under solar light, TiO_2_‐WO_3‐x_ NCs demonstrated an increased photocatalytic activity relative to the original TiO_2_ seeds. A lower conversion is obtained by W_x_Ti_1‐x_O_2_ NCs compared to both heterostructures and bare TiO_2_. The faster oxidative action of TiO_2_‐WO_3‐x_ NCs can further be inferred by investigations of the time required to convert 30 % of the initial alcohol amount (Table 1, t_x = 30_) and from the variation of 4‐MBA concentration over irradiation (**Figure** **5**). One hundred minutes are required for TiO_2_‐WO_3‐x_ to convert 30 % of 4‐MBA, while W_x_Ti_1‐x_O_2_ takes more than twice that time to reach a similar percentage. Accordingly, nanosized bare TiO_2_ seeds resulted in a higher oxidation rate than the widely employed P25, which has been reported to be the most photoactive commercial TiO_2_ sample. Interestingly, the presence of W heteroelement, as either dopant in the ternary nanostructure or as WO_3‐x_ segregated entity, revealed beneficial in terms of selectivity control toward 4‐anisaldehyde (Table 1, S_AA_ t = 4 h), whereas both TiO_2_ samples, regardless of their synthetic origin, provided similar, less‐selective outcomes. Indeed, a slight selectivity increase is determined in the case of TiO_2_‐WO_3‐x_, whereas it is almost doubled for W_x_Ti_1‐x_O_2_. This trend is wholly confirmed by evaluating the selectivity toward 4‐anisaldehyde at 30 % of alcohol consumption (Table 1, S_AA_ x = 30); higher percentages are achieved relative to both TiO_2_ samples, although different irradiation times are required (Table 1, t_x = 30_). These findings are better highlighted by analyzing the yield of anisaldehyde (Table 1, Y_AA_ t = 4 h), where the highest values are obtained in the presence of heterostructures. The addition of W also positively impacts the formation of 4‐methoxybenzoic acid, achieving a selectivity of ≈3.5 % by using bare TiO_2_, ≈5% with W_x_Ti_1‐x_O_2,_ and up to 10 % with the heterostructures. The difference in selectivity observed between the two samples containing W is likely attributable to the difference in surface properties of the two photocatalysts. In the case of TiO_2_‐WO_3‐x_, the WO_3‐x_ domains on the TiO_2_ surface may inhibit the decomposition of aldehyde, which aligns with findings in the literature.^[^ ^91^, ^92^, ^93^ ^]^

**Table 1:** Liquid‐phase 4‐MBA partial oxidation results. X, S, and Y, respectively, represent conversion, selectivity, and yield after 4 h of simulated solar light irradiation. S_x = 30_ selectivity at 30 % of conversion, t_x = 30_ time to reach 30 % of conversion. Corresponding formulae are indicated in the table. C_0_=initial concentration of reactant; C_r_=concentration of the reactant at a certain reaction time; C_p_=concentration of the target product. The AA subscript relates to anisaldehyde. The corresponding QE values are listed in the final column.

SOLID CATALYST	X_t=4 h_ $[\frac{C_{0}-C_{r}}{C_{0}}]\times 100$	S_AA t=4 h_ $[\frac{C_{p}}{C_{0}-C_{r}}]\times 100$	Y_AA t=4 h_ $[\frac{C_{p}}{C_{0}}]\times 100$	S_Acid_ $[\frac{C_{p}}{C_{0}-C_{r}}]\times 100$	S_AA x=30_ $[\frac{C_{p}}{0.7C_{0}}]\times 100$	t_x=30_ [min]	QE [%]
**Simulated solar light irradiation**							
**TiO_2_ seeds**	49	19	9	3	22	120	7.8
**W_x_Ti_1‐x_O_2_**	30	41	12	5	41	240	2.3
**TiO_2_‐WO_3‐x_**	64	24	15	10	40	100	10.1
**TiO_2_ commercial (P25)**	41	19	7	4	21	152	3.9

To qualitatively assess the contribution of the vis‐NIR/plasmonic absorbance to photocatalytic activity, specific photoconversion experiments of 4‐MBA are also performed under pure UV irradiation for 4 h. The percentage of 4‐MBA converted with TiO_2_‐WO_3‐x_ nanoheterostructure remained higher under sunlight irradiation (64% vs 60% after 4 h irradiation, see Table 1; Table S3, the latter in the Supporting Information), despite under pure UV irradiation, the power density was higher (100 W m^−2^ vs 38 W m^−2^ for solar light). This provides experimental evidence that the vis‐NIR portion of the solar spectrum, not merely photon flux, drives the enhanced photoactivity. The increase in photoconversion efficiency under vis‐NIR can be attributed to a combination of hot electron‐assisted photocatalysis^[^ ^94^ ^]^ and photothermocatalysis^[^ ^95^, ^96^, ^97^ ^]^ generated in the WO_3‐x_ domain through intraband excitation. The home‐prepared samples demonstrate superior and increasing selectivity for aldehyde and a significant conversion degree even under UV light.

The quantum efficiency (QE) values are listed in Table 1 and Table S3 (Supporting Information). These QE values were determined in accordance with the IUPAC guidelines.^[^ ^92^, ^93^ ^]^ For further details, please refer to the Supporting Information. The QEs relative to solar light irradiation (Table 1) are higher than those measured under high‐intensity pure UV light (Table S3, Supporting Information). Under solar light, which includes visible wavelengths and has a much lower UV flux, the heterostructure achieves a high QE of 10.1%. The significant increase in efficiency—from 0.070% under UV irradiation—demonstrates that the TiO_2_ / WO_3‐x_ interface effectively separates the photogenerated charges and optimally utilizes the visible light spectrum. In contrast, the ternary material, while performing best under UV conditions, experiences a decline in performance, recording the lowest QE of all samples at 2.3%. This indicates that the defects introduced by W‐doping, which provide a slight benefit in high‐energy UV, become dominant charge recombination centers under the broader, lower‐intensity solar spectrum. Under high‐intensity, high‐energy UV irradiation, the overall QE values for all samples are extremely low. These low QE values indicate that the entire reaction system is operating under photonic saturation.^[^ ^98^, ^99^ ^]^ Consequently, the majority of charges recombine, and the overall kinetic rate is limited by mass transfer of reactant across the surface of the photocatalyst. In this saturated regime, W_x_Ti_1‐x_O_2_ achieves the highest QE. This minor advantage suggests that the W‐doping creates defects that are particularly effective at trapping charges when hit by high‐energy UV photons. TiO_2_‐WO_3‐x_ performs similarly, indicating that the benefits of the heterojunction are negated when the system operates at maximum recombination rate.

The effectiveness of separating photo‐generated charge carriers, the formation of intra‐gap states, the occurrence of hot carriers in the plasmonic WO_3‐x_ domain, and the dynamic acceptor‐donor interactions likely happening on the photo‐catalyst substrate are key mechanisms that collectively or individually influence the photo‐activity process. Oxidation is expected to occur through a direct and/or indirect reaction path, depending on whether the process is straightforwardly mediated by holes at TiO_2_ nanointerfaces or indirectly by hydroxyl (H_2_O‐assisted) or superoxide radical species or hydrogen peroxide diffusing in the liquid medium.^[^ ^100^, ^101^ ^]^ Experimental results under solar light evidenced a pronounced photo‐oxidant activity in the case of heterostructured morphologies. The UV portion of the spectrum makes the photogenerated holes available for oxidation in the TiO_2_ nanodomains and much less prone to recombination because of effective spatial charge delocalization and increased charge carrier lifetimes (see photoluminescence spectra in Figure S14a, Supporting Information).^[^ ^102^ ^]^ Excitation within the domain of the plasmon peak range promotes the generation of hot carriers, which, due to their higher energies, can generate other effective radical species, such as superoxide anion, or exhibit high oxidizing efficacy.^[^ ^94^, ^103^ ^]^ Additionally, a photothermal effect due to a NIR‐assisted plasmonic heating is expected.^[^ ^95^, ^97^ ^]^ Indeed, as observed by ultrafast pump‐probe spectroscopic experiments, hot carriers rapidly thermalize with the crystal lattice (via electron‐phonon and phonon‐phonon scattering) within picoseconds, thus converting the absorbed light energy into highly localized heat at the surface of the WO_3‐x_ domain. In the case of W_x_Ti_1‐x_O_2_ NCs, as already demonstrated in the literature, the inclusion of heteroatoms generates intra‐gap energy states that can act as trap states for electrons.^[^ ^44^, ^50^, ^102^, ^104^ ^]^ However, this suggests that introducing or coupling W into/with the TiO_2_ matrix (W_x_Ti_1‐x_O_2_ and TiO_2_‐WO_3‐x_ respectively) and slowing down the photo‐oxidation reaction, enhances the selectivity toward anisaldehyde; this observation aligns with the literature that generally shows that selectivity increases as conversion decreases.^[^ ^14^, ^105^ ^]^ This finding is a general behavior due to the competition on the photocatalyst surface between the produced molecules and the original substrate. A more direct comparison of the samples used is reported in Figure S14b (Supporting Information).

**Figure 6:**
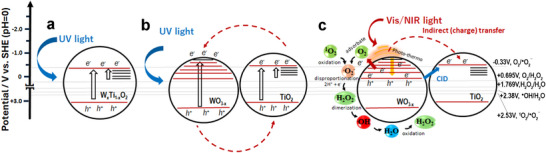
UV‐ and vis‐NIR‐activated electronic pathways for W_x_Ti_1‐x_O_2_ (a) and TiO_2_‐WO_3‐x_ (b,c) NCs. CID stands for chemical interface damping. Redox potentials of reactive oxygen species that may be critical for the oxidation of organics are reported.

According to ultrafast spectroscopy results, the dynamics of the involved charge carriers can be explained as follows: in the case of heterostructures and vis‐NIR excitation, high‐energy hot electrons and holes (the latter of low oxidizing capacity) are generated, both localized in the CB of the WO_3‐x_ plasmonic domain. Highly energetic hot carriers can transfer with the mechanisms discussed above (direct or indirect transfer). Given the minute thickness, many of these charges reside on the surface of the plasmonic domain, likely functioning as efficient charge injectors and thereby enhancing chemical processes. In this case, the electron energy could reduce O_2_ to superoxide anions (single‐electron reduction)^[^ ^94^, ^101^ ^]^ or to hydrogen peroxide (two‐electron reduction).^[^ ^101^ ^]^ Also, ultrafast spectroscopic experiments clearly show hot carrier relaxation through electron‐phonon scattering (heat transfer to the lattice) and then, on a timescale of ps, phonon‐phonon scattering (lattice cooling and dissipation of heat into the environment). We therefore expect a NIR‐assisted photothermalcatalytic contribution.^[^ ^95^, ^97^, ^106^ ^]^ On the contrary, in the event of UV excitation, different players are involved; electrons and holes form in the CB and VB of TiO_2,_ respectively, while hot electrons form in the CB of WO_3‐x_ and hot holes in the VB of WO_3‐x_. The UV contribution involves many different charge carriers, particularly including highly oxidizing holes. In the case of W_x_Ti_1‐x_O_2_, which is only active in the UV range, electrons will form in the CB and holes in the VB. In this other case, it is reasonable to assume that electrons with a potential less negative than the bottom of the CB of the pure TiO_2_ can promote O_2_ reduction and H_2_O_2_ formation, but are less likely to reduce O_2_ to form superoxide anions. Therefore, they can facilitate oxidation by a two‐electron process, i.e., via H_2_O_2_.^[^ ^101^ ^]^ This helps rationalize their higher selectivity and lower efficiencies. Additionally, energetic holes can oxidize H_2_O either to H_2_O_2_ (two‐hole oxidation) or to the hydroxyl radical (single‐hole oxidation).^[^ ^94^, ^101^ ^]^ **Figure** **6** illustrates the possible carrier paths as described.

The comparative analysis between the two tungsten‐modified architectures highlights their suitability for different light sources. The data reveal that the two materials are optimized for completely different irradiation regimes, confirming the superior design of the heterostructure for solar energy. The latter can be interpreted as the result of the combination of a photocatalytic effect due to hot carriers that may generate the charges necessary for the surface reaction, and of a photothermocatalytic effect that can provide the thermal energy necessary to accelerate the kinetics of the surface reaction and mitigate the diffusion limitations. Both simultaneously contribute to determining the considerable oxidizing action toward 4‐MBA.

## 3 Conclusion

Nanosized W_x_Ti_1‐x_O_2_ and heterostructured TiO_2_‐WO_3‐x_ NCs have been developed in a hydroalcoholic solution using MW to deliver energy to the reagents. The two colloidal nanomaterials have been obtained through a one‐pot or a seeded‐growth procedure, respectively. The chemical nature of the reaction mixture guarantees a swift response to MW, and the quick in‐core heating makes the reagents promptly available to promote the anisotropic growth of WO_3‐x_ in the heterostructures. In all cases, very few minutes are enough for the product to be delivered, and the employment of a modular apparatus enables the production of several tens of grams of each sample, which remains stable in pure water for months. The formation of intermixed‐metal chloroisopropoxide species is called upon to explain the nucleation of ternary nanostructures, a claim supported by literature evidence. Whereas, concerning the nucleation and growth of heterostructures and specifically the formation and development of a new phase onto a preexisting one, we provide the following experimental observations: i) thermodynamic conditions (interphase lattice mismatch and/or defect points onto the seeds working as nucleation germs) promote heterogeneous nucleation in place of a homogeneous route; ii) the hydroxyl groups‐based chemical nature of the seed surface initiates the hetero‐nucleation stage in the presence of TMAH, promoting the nucleophilic attack of tungsten precursor;^[^ ^57^ ^]^ iii) the polar character of the seed surface contributes to localizing right there MW (over)heating because of a dielectric loss mechanism of interfacial polarization triggered by hydroxyl groups;^[^ ^107^, ^108^, ^109^ ^]^ iv) the seed's ability toward MW absorption is further improved by the addition of W precursor that before forming elongated nanostructures creates around the seed a thin and discontinuous shell which promotes a loss mechanism of interfacial polarization through heterointerface engineering;^[^ ^110^ ^]^ v) kinetic growth is driven by a large availability of tungsten precursors at high MW‐induced temperatures. All of these factors, either collectively or individually, can account for the heterogeneous nucleation and the anisotropic growth of the WO_3‐x_ phase.

While the ternary material demonstrates activity and notable selectivity exclusively under UV excitation, heterostructures offer interesting functionality, particularly in enhancing light absorption within the NIR spectral range. Photocatalytic performances have benefited from this in terms of efficiency and selectivity toward alcohol‐to‐aldehyde partial oxidation in aqueous solution. Because of its unique electronic structure, TiO_2_‐WO_3‐x_ is effective across a broad spectral range from UV to NIR, generating hot carriers in all scenarios. NIR photoexcitation involves plasmon excitation, which significantly modulates photocatalytic activity. This effect is notable in TiO_2_‐WO_3‐x_ but insignificant in W_x_Ti_1‐x_O_2_ NCs. Determining whether NIR‐assisted hot electron transfer occurs from the plasmonic domain to the semiconducting domain (or molecules) based solely on direct reactivity enhancement is quite challenging and exceeds the scope of this work. The nearly degenerate bandgaps of the two materials hinder the possibility of performing detection‐frequency‐resolved pump‐probe experiments in the vis‐NIR to observe a clear transfer. One possible strategy would involve using time‐resolved terahertz spectroscopy for a direct investigation of the dynamics of the intraband states in the CB of the WO_3‐x_ domain. Additionally, since catalysis is thermally activated, it is important to consider the potential impact of NIR‐assisted temperature increase in the lattice and the surface due to plasmonic photothermal effects.

The developed nano‐catalysts successfully fulfill the essential criteria of catalysis science—activity, selectivity, and stability. All the samples work well for the selective oxidation of 4‐MBA, particularly under simulated solar light irradiation. However, the TiO_2_‐WO_3‐x_ heterostructured NCs stand out as particularly effective examples in the field of plasmonic photocatalysis and light‐harvesting. Their enhanced performance is attributable to the synergistic combination of the following factors: i) a significant efficiency of charge separation at the heterojunction interface; ii) an enhancement of the vis‐NIR absorption cross‐section resulting from the plasmonic domain in intimate contact with the semiconducting component; iii) a wide interfacial area that concentrates the light electric field near the plasmonic‐semiconductor junction; iv) an extensive surface area of the metallic component optimizing reaction sites, and v) the availability of hot carriers across a wide range of excitation wavelengths, maximizing the photocatalytic potential.

## 4 Experimental Section

### Chemicals

All chemicals were used as received without further purification. The following chemicals were purchased from Sigma–Aldrich: tetramethylammonium hydroxide pentahydrate (CH_3_)_4_N(OH)·5H_2_O (TMAH, ≥ 97 %), titanium (IV) isopropoxide Ti[OCH(CH_3_)_2_]_4_ ((Ti(OPr^i^)_4_)), 97 %), tungsten hexachloride WCl_6_ (≥ 99.9 %), anhydrous 2‐propanol (CH_3_)_2_CHOH. Stock solutions of titanium and tungsten precursors in anhydrous 2‐propanol were prepared in a glovebox at room temperature.

### Synthesis of TiO_2_ Seeds

TiO_2_ NCs were prepared according to a previously reported procedure with minor changes.^[^ ^57^ ^]^ Briefly, 140 mg (0.77 mmol) of TMAH were dissolved in 50 mL of Milli‐Q water (18.2 MΩ cm^−1^) and loaded into a 100 mL three‐neck flask. Next, under nitrogen flow, the solution was cooled to 0°C by placing the flask in a large crystallizing dish filled with an ice/water mixture. At this point, a solution of 0.6 mL (2 mmol) of Ti(OPr^i^)_4_ in 3.4 mL of anhydrous 2‐propanol was added dropwise while the mixture was stirred vigorously. After the addition was complete, the mixture was left to stir for an additional 15 min and then transferred into a Teflon vessel for MW heating. The vessel was sealed and exposed to intense MW irradiation (MW digestion system, Mars, CEM, Matthews, NC). The reaction mixture was microwaved at 180°C for 180 min (heating power 800 W). The resulting milky suspension was used as synthesized for the further growth of the WO_3_ domain; no additional purification step was needed.

### Synthesis of Ternary W_x_Ti_1‐x_O_2_ NCs

To synthesize W_x_Ti_1‐x_O_2_ NCs, a solution of 1.5 mmol of TMAH in 150 mL of Milli‐Q water was loaded into a 250 mL three‐neck flask and, under nitrogen flow, cooled to 0°C by placing the flask in a large crystallizing dish filled with an ice/water mixture. Then, a mixture of titanium and tungsten precursors (that was, 3.2 mmol of Ti(OPr^i^)_4_ and 0.15 mmol of WCl_6_ in 64 mL of anhydrous 2‐propanol) was added dropwise via a syringe pump (rate 0.2 mL min^−1^) while the mixture was stirred vigorously. After the addition was complete, the mixture was left to stir for an additional 15 min and then transferred into a Teflon vessel for MW heating. The vessel was sealed and exposed to intense MW irradiation. The reaction mixture was microwaved and maintained at 180°C for 180 min. The resulting solution was purified from unreacted reagents by washing steps (three times) with Milli‐Q water on centrifuge filters Amicon Ultra‐15, Molecular Weight cut‐off 50 kDa.

### Synthesis of TiO_2_‐WO_3‐x_ Heterostructured NCs

Colloidal TiO_2_‐WO_3‐x_ heterostructured NCs were prepared by hydrothermal treatment of preformed TiO_2_ seeds in the presence of tungsten precursor. In a typical synthesis, 25 mL of TiO_2_ seed solution ([Ti]^4+^ = 0.014 mm calculated on titanium elemental content from ICP analysis) were loaded into a 50 mL three‐neck flask and, under nitrogen flow, cooled to 0°C by placing the flask in a large crystallizing dish filled with ice/water mixture. At this point, a solution of 40 mg (0,10 mmol) of WCl_6_ dissolved in 8 mL of anhydrous 2‐propanol was added dropwise (rate 0.2 mL min^−1^), while the mixture was stirred vigorously. After that, the mixture was left to stir for an additional 15 min and then transferred into a Teflon vessel for MW heating. The vessel was sealed and exposed to high‐intensity MW irradiation. The reaction mixture was microwaved at 180°C for 10 min. The resulting solution was purified from unreacted reagents by washing steps (three times) with Milli‐Q water on centrifuge filters Amicon Ultra‐15, Molecular Weight cut‐off (MWCO) 50 kDa. The time of growth was extended to 180 min without detecting any significant change in the outcome. The WO_3‐x_ NCs synthetic procedure has already been reported elsewhere.^[^ ^58^, ^111^ ^]^

*Composition Analysis of* *NCs*: Elemental analysis was carried out via inductively coupled plasma atomic emission spectroscopy (ICP‐AES), using a Varian Vista AX Spectrometer. The samples were digested in concentrated HF/HCl (0,1:1, v:v). Specifically, 1 mL of concentrated HCl (37 %, w/w) was loaded into 50 µL of sample solution and left overnight. Therefore, 100 µL of HF (38 %, w/w) were added, and the solution was sonicated in the ultrasonic bath for 2 h at 60°C to achieve the complete sample mineralization. Samples were properly diluted and filtered over a 0.2 µm PTFE syringe filter before being injected into the ICP.

### Structural Characterization by TEM/STEM

Low‐magnification TEM images were recorded on a Jeol JEM 1400 Plus microscope operating at 120 keV. The samples were prepared by drop‐casting a dilute solution of purified NCs onto carbon‐coated copper grids.

### High‐Resolution Electron Microscopy Analyses

High‐resolution TEM as well as Annular Dark Field Scanning Transmission Electron Microscopy (ADF‐STEM) were performed using Jeol 2010F (200 keV) and FEI TITAN ETEM (80‐300 keV) operating in a vacuum. The samples were mashed in a mortar and then dispersed in ethanol under sonication for 15 min. Then, a droplet from the surface of the suspension was loaded on a 300‐mesh copper grid for microscopy. After drying, the grid was set on a specific, simple tilt microscopy holder.

*Diffuse Reflectance Spectroscopy*: Diffuse reflectance spectra were recorded with a Shimadzu UV‐2401 PC spectrophotometer in the 200‐800 nm wavelength range, by using BaSO_4_ as the reference material. Bandgap values were determined by plotting the modified Kubelka–Munk function, [F(R’∞)hν]^1/2^, versus the energy of the exciting light.^[^ ^71^, ^112^ ^]^

*Specific Surface Area*: The specific surface area (SSA) of the powders was measured by a FlowSorb 2300 instrument (Micromeritics) by using the single‐point BET method.^[^ ^113^ ^]^ Before the measurements, the samples were degassed by a N_2_/He mixture 30/70 (v/v) for 0.5 h at 523 K.

*Raman Spectroscopy*: Raman spectra were recorded by a BwTek i‐Raman plus system equipped with a 785 nm laser focused on the sample through a microscope equipped with a 20x magnification lens. The signal integration time was 5 s, and every spectrum was an average of 8 repetitions.

*X‐ray Photoelectron Spectroscopy*: X‐ray photoelectron spectroscopy analyses were performed through a VG Microtech ESCA 3000 Multilab, equipped with a dual Mg/Al anode by using the Al Kα source (1486.6 eV) at 14 kV and 15 mA. The sample powders were fixed on a double‐sided adhesive tape, and the same powder amount was used for all of the samples. Survey spectra and individual peak regions were acquired at 50 and 20 eV pass energy, respectively, and the pressure in the analysis chamber was in the range of 10^−8^ Torr. All the binding energies were calibrated against the C 1s peak energy set at 285.1 eV, arising from adventitious carbon. The fitting of the spectra was performed with the software provided by VG. The binding energy values were quoted with a precision of ± 0.15 eV.

*X‐ray Diffraction*: X‐ray diffraction measurements were performed with a PANalytical Empyrean X‐ray diffractometer equipped with a 1.8 kW Cu Kα ceramic X‐ray tube and a PIXcel3D 2 x 2 area detector, working at 45 kV and 40 mA, recorded under ambient conditions with the parallel beam geometry and symmetric reflection mode. Samples were prepared by casting multiple drops of NC solution onto a zero‐diffraction silicon substrate and analyzed after water evaporation.

### Photoluminescence Measures

Photoluminescence (PL) spectra were acquired by a JASCO FP‐8550 spectrofluorometer in emission mode, covering a wavelength range of 350–800 nm with an excitation wavelength of 320 nm.

### Photoreactivity Experiments

The commercial TiO_2_ Aeroxide P25 was used for comparison due to its widespread use and high photocatalytic activity. The photocatalytic activity of the samples was evaluated in aqueous solution. The values reported in the manuscript were the averaged results from two separate runs, with an experimental error of ≈3–4%.

### 4‐Methoxybenzyl Alcohol Partial Oxidation

4‐Methoxybenzyl alcohol (4‐MBA) was used as a probe molecule in the liquid phase. Its partial oxidation was carried out in a Pyrex cylindrical reactor containing 150 mL of 0.5 mm aqueous solution of the alcohol. An internally placed 100 W halogen lamp (radiant power ≈3.9 W m^−2^ and ≈38 W m^−2^ in the 450–750 nm and 315–400 nm ranges, respectively) was used as the irradiation source to simulate the solar light spectrum. Runs were also carried out with a 125 W medium‐pressure Hg lamp (radiant power of 100 W m^−2^). The experiments lasted 4 h, and during their carrying out, the reactor was open to atmospheric air, allowing the dissolution of O_2_ in the aqueous suspension. A thimble around the reactor allowed for the circulation of water and the maintenance of the reaction temperature at ≈28°C. During the runs, samples of the irradiated suspension were withdrawn every 30 min and filtered through 0.25 µm membranes (PTFE, Whatman) to separate the photocatalyst particles. A Beckman Coulter HPLC instrument equipped with a Diode Array detector was used to determine the concentration of the substrate and its oxidation products. A Phenomenex KINETEK 5 µm C18 100A column (4.6 mm × 150 mm) was used, and the eluent (0.8 mL min^−1^) consisted of a mixture of acetonitrile and 13 mm trifluoroacetic acid aqueous solution (20:80, v:v). Standards (purity > 99 %) to identify the different compounds and to obtain the calibration curves were obtained from Sigma–Aldrich. The intermediates detected were 4‐anisaldehyde (AA) or 4‐methoxybenzyl aldehyde and 4‐methoxybenzoic acid. The quantum efficiency of the reaction was calculated following the guidelines set by IUPAC.^[^ ^92^ ^]^

### Pump‐Probe Spectroscopy

The ultrafast differential transmission measurements have been performed by using an amplified femtosecond Ti: sapphire laser (Coherent Libra). The fundamental beam was centered ≈800 nm with a repetition rate of 2 kHz and a temporal pulse duration of ≈100 fs. The third harmonic of the laser output used to study the interband transition of the different systems has been generated as a sum of the fundamental and its second harmonic in a β‐barium borate (BBO) crystal. Non‐collinear optical parametric amplifier (NOPA) built as in Ref. [114] has been used to generate < 20 fs pulses centered in the visible and NIR spectral range. To generate a probe pulse, a small portion of the fundamental beam was focused on a 2 mm CaF_2_ plate that generates a broadband pulse in the range of 330–700 nm. To prevent any degradation of the crystal, it was kept moving with an electronic rotator. The probe beam was then detected with a spectrometer equipped with a 1024 Si pixel array. By recording pump‐on and off spectra, the differential transmissivity signal was extracted as a function of pump‐probe delay and wavelengths. In differential transmissivity pump‐probe experiments, a positive signal was related to an increase in the transmittivity, therefore to a photo‐bleaching of the direct transitions. Negative signals were instead a consequence of a decrease in the transmittivity, linked to photo‐induced absorption. Cross‐phase modulation occurs when an ultrashort pulse interacts with a glass substrate. This was a consequence of the non‐resonant Kerr effect when modulation of the non‐linear component of the refractive index leads to a spectral shift of the probe pulses, whose duration depends on the pump pulses’ temporal width. This can be removed in a post‐processing step. The absence of power exchange between the pump and the glass allows the removal of the artifact from the actual relaxation dynamics, following several methods.^[^ ^115^ ^]^ Here, the method proposed by Bresci et al. was used, fitting the artifact as a sum of a Gaussian and its first derivative.^[^ ^116^ ^]^

## Conflict of Interest

The authors declare no conflict of interest.

## Author Contributions

R.S. and M.G. contributed equally to this work and are co‐first authors in this work. A.F., A.G., and L.C. jointly supervised and are co‐last authors in this work. A.F., R.S., and L.C. conceived the idea and designed the experiments. A.F., E.P., S.F.O., and R.S. carried out all the syntheses and the low‐magnification TEM, structural, and conventional optical characterization. C.F. and L.R. carried out the HRTEM characterization. C.N. performed the analysis, editing, and interpretation of the HRTEM images; M.B., A.M.V., and L.P. performed photocatalytic, Raman, and XPS measurements. M.G., A.G., and G.C. conceptualized and carried out the pump‐probe measurements, performed the analysis, editing, and discussion of related data. L.C. revised the overall manuscript. All the authors wrote and revised drafts and contributed to the scientific discussion of the article.

## Supporting information



Supporting Information

## Data Availability

The data that support the findings of this study are available from the corresponding author upon reasonable request.
