# Supplementary material for: Scalable, Microwave‐Enabled Synthesis of Ternary WxTi1‐xO2 and Heterostructured TiO2‐WO3‐x Colloidal Nanocrystals: Carrier Dynamics and Photocatalytic Properties
Source: Adv Sci (Weinh). 2025 Dec 16;13(11):e14916. doi: 10.1002/advs.202514916 (PMC12931172; doi:10.1002/advs.202514916)
Supplement: Supplementary file 1 — Supporting Information [file ADVS-13-e14916-s001.docx]

Supporting information

Scalable, Microwave-Enabled Synthesis of Ternary W_x_Ti_1-x_O_2_ and Heterostructured TiO_2_-WO_3-x_ Colloidal Nanocrystals: Carrier Dynamics and Photocatalytic Properties

*Riccardo Scarfiello^§^, Michele Guizzardi^§^, Angela Fiore*, Armando Genco*, Concetta Nobile, Elisabetta Perrone, Sara Fernanda Orsini, Clémentine Fellah, Lucian Roiban, Marianna Bellardita, Anna Maria Venezia, Giulio Cerullo, Leonardo Palmisano, Luigi Carbone**

Dr. R. Scarfiello, Dr. C. Nobile, Mrs. E. Perrone, Dr. S.F. Orsini, and Dr. L. Carbone

CNR NANOTEC – Institute of Nanotechnology c/o Campus Ecotekne, University of Salento, via Monteroni, Lecce 73100, Italy

E-mail: [luigi.carbone@cnr.it](mailto:luigi.carbone@cnr.it)

Dr. R. Scarfiello, Dr. L. Carbone

Istituto Italiano di Tecnologia, Center for Biomolecular Nanotechnologies, Arnesano (LE) 73010, Italy

Dr. Angela Fiore

Agenzia Nazionale per le nuove tecnologie, l'energia e lo sviluppo economico sostenibile ENEA-SSPT-PROMAS-MATAS, Centro Ricerche Brindisi, c/o Cittadella della Ricerca S.S.7 "Appia" Km 706, 72100 Brindisi, Italy

E-mail: [angela.fiore@enea.it](mailto:angela.fiore@enea.it)

Dr. C. Fellah

Université de Lyon, ENS Lyon, Université Lyon 1, CNRS, LGL-TPE, F-69007 Lyon, 6 France

Dr. L. Roiban

Université de Lyon, INSA Lyon - Institut National des Sciences Appliquées de Lyon, MATEIS UMR, CNRS 5510, 21 Avenue Jean Capelle, 69625 Villeurbanne, France

Prof. M. Bellardita, Prof. L. Palmisano

Dipartimento di Ingegneria, Università di Palermo, ed. 6, Viale delle Scienze, 90128 Palermo, Italy

Dr. A.M. Venezia

ISMN-CNR, Via Ugo la Malfa 153, 90146, Palermo, Italy

Dr. A. Genco, Dr. M. Guizzardi, Prof. G. Cerullo

Dipartimento di Fisica, Politecnico di Milano, Piazza Leonardo da Vinci, 32, 20133, Milano, Italy

E-mail: [armando.genco@polimi.it](mailto:armando.genco@polimi.it)

Keywords: defective oxide heterostructures, ternary oxide, microwave synthesis, hot carrier dynamics, photocatalysis

__________________________________________________________________________

Contents

Figure S1. From left to right, low-resolution TEM, ADF-STEM in SEM, and SEM overviews of (a-c) W_x_Ti_1-x_O_2_; (d-f) starting TiO_2_ seeds; (g-i) TiO_2_-WO_3-x_. All scale bars are 20 nm.

Figure S2. Representative TEM pictures of TiO_2_-WO_3-x_ heterostructures prepared at different Ti:W molar ratios. The Ti to W ratios reported over each image have been determined via ICP-AES analysis. Instead, the synthetic amounts of WCl_6_ injected within the same amount of TiO_2_ seeds (details in the experimental section) of each synthetic process are respectively: (a) 25 mg, (b) 50 mg, (c) 75 mg, (d) 100 mg in 8 ml anhydrous 2-propanol.

Figure S3. Representative TEM pictures of TiO_2_-WO_3-x_ heterostructures as obtained by scaling up 3 times a typical synthesis program as described in the experimental section. All scale bars are 50 nm.

Figure S4. (a,c) SEM and (b,d) in-SEM ADF-STEM overviews, recorded on a TEM grid, of TiO_2_-WO_3-x_ heterostructures as obtained by tripling the amounts of the reagents of a typical synthesis recipe described in the experimental section. All scale bars are 20 nm.

Figure S5. Effects upon hetero-structuring of Ti:W precursor molar ratio and of TiO_2_ seeds purification. Concerning the Ti-to-W molar ratios, the number of WO_3-x_ rod-like domains for each TiO_2_ seed resulted significantly dependent on the employed precursors’ molar ratios. Images report three different examples of heterostructures prepared following the same reaction conditions, however, at different Ti:W precursor molar ratios reported respectively in the image. Furthermore, the panel shows the effectiveness of tungsten precursors toward heterogeneous nucleation when occurring onto purified or unwashed TiO_2_ seeds.

Figure S6. Comparison of heterostructured samples of TiO_2_-WO_3-x_, either obtained by the use of MW irradiation or conventional convective heating using a heating mantle or both approaches in sequence or autoclave; all the samples have been produced with a constant Ti:W precursor molar ratio of 4:1. (a) Sample refluxed at 100°C for 3h by heating mantle; (b) sample heated by MW irradiation at 100°C for 3h. Picture (c) shows TiO_2_-WO_3-x_ heterostructures obtained by further heating sample (a) at 180°C for 10 min through MW. Images (d-f) report samples developed at 180°C within an autoclave with Ti:W precursor molar ratio of 4:1 and (g-i) with constant Ti:W precursor molar ratio of 1:1, for a time growth of 30 min, 3 h, and 24 h, respectively.

Figure S7. High-resolution ADF-STEM images of TiO_2_-WO_3-x_ nanocrystals. All scale bars are 20 nm.

Figure S8. HRTEM and ADF-STEM of heterostructures, evidencing the existence of very few layers of tungsten oxide, plausibly amorphous, wrapping the TiO_2_ domain.

Figure S9. (a) XRD pattern of W_x_Ti_1-x_O_2_, TiO_2_-WO_3-x_, and TiO_2_ pristine seeds. (b) XPS of W_x_Ti_1-x_O_2_ and TiO_2_-WO_3-x_. (c-d) Complete Raman spectra of W_x_Ti_1-x_O_2_ and TiO_2_-WO_3-x_ NCs, TiO_2_ seeds, and TiO_2_ P25 commercial references for comparison. (e) 1-Eg zoom mode at 144.5 cm-1 of TiO_2_ seeds, W_x_Ti_1-x_O_2_ and TiO_2_-WO_3-x_ NCs and TiO_2_.

Figure S10. (a) DR spectra of MW-developed samples, namely TiO_2_ seeds, W_x_Ti_1-x_O_2_, and TiO_2_-WO_3-x_ NCs, and commercial references. (b) Energy band gaps derived from the Kubelka-Munk treatment. (c) Extinction spectra of corresponding samples dispersed in aqueous media. (d) Extinction optical features in aqueous media and (e-h) corresponding TEM images of samples prepared at different Ti:W molar ratios; the same color code is adopted for optical spectra reported in picture (d) and TEM image frames.

Table S1. W%-related content determined by ICP-AES analysis. Values of energy band gaps (last column) are also reported, obtained from the Kubelka-Munk function performed through optical analysis.

Table S2. XPS data of developed W_x_Ti_1-x_O_2_ and TiO_2_-WO_3-x_ NCs and of pristine TiO_2_ seeds.

**Figure S11.** Baseline approach to evaluate the bandgap energy from the Tauc plot as reported by Macyk et. al.^[1]^.

**Figure S12.** Pump-probe transient reflectivity maps exciting the samples in the near-infrared (800nm) for different systems: (a) TiO_2_-WO_3-x_, (b)WO_3-x_, (c) TiO_2_, (d) Ternary (W_x_Ti_1-x_O_2_). Only TiO_2_-WO_3-x_ and WO_3-x_ show a negative-positive signal persistent at longer times compared to the pulse duration, attributed to the plasmonic response. The strong and ultrashort negative feature present in all the maps at about 650 nm is attributed to coherent Raman scattering of the water solvent.

Figure S13. (a-d) Pump-probe transient transmission maps exciting the plasmon resonance of the heterostructure with NIR pulses tuned at 1000 nm, compressed to < 20 fs for TiO_2_-WO_3-x_ heterostructures with a Ti/W content ratio of 4.33 (a), 2.31 (b), 1.61 (c), 1.08 (d), respectively.

Table S3. Liquid-phase 4-MBA partial oxidation results. X, S, and Y, respectively, represent conversion, selectivity, and yield after 4 h of UV irradiation. The corresponding QE values are listed in the final column.

Figure S14. (a) Photoluminescence spectra of the different samples (λ_excitation_ = 320 nm). (b) 4-MBA conversion, selectivity, and yield to the corresponding aldehyde and acid after 4 h of simulated solar light irradiation.


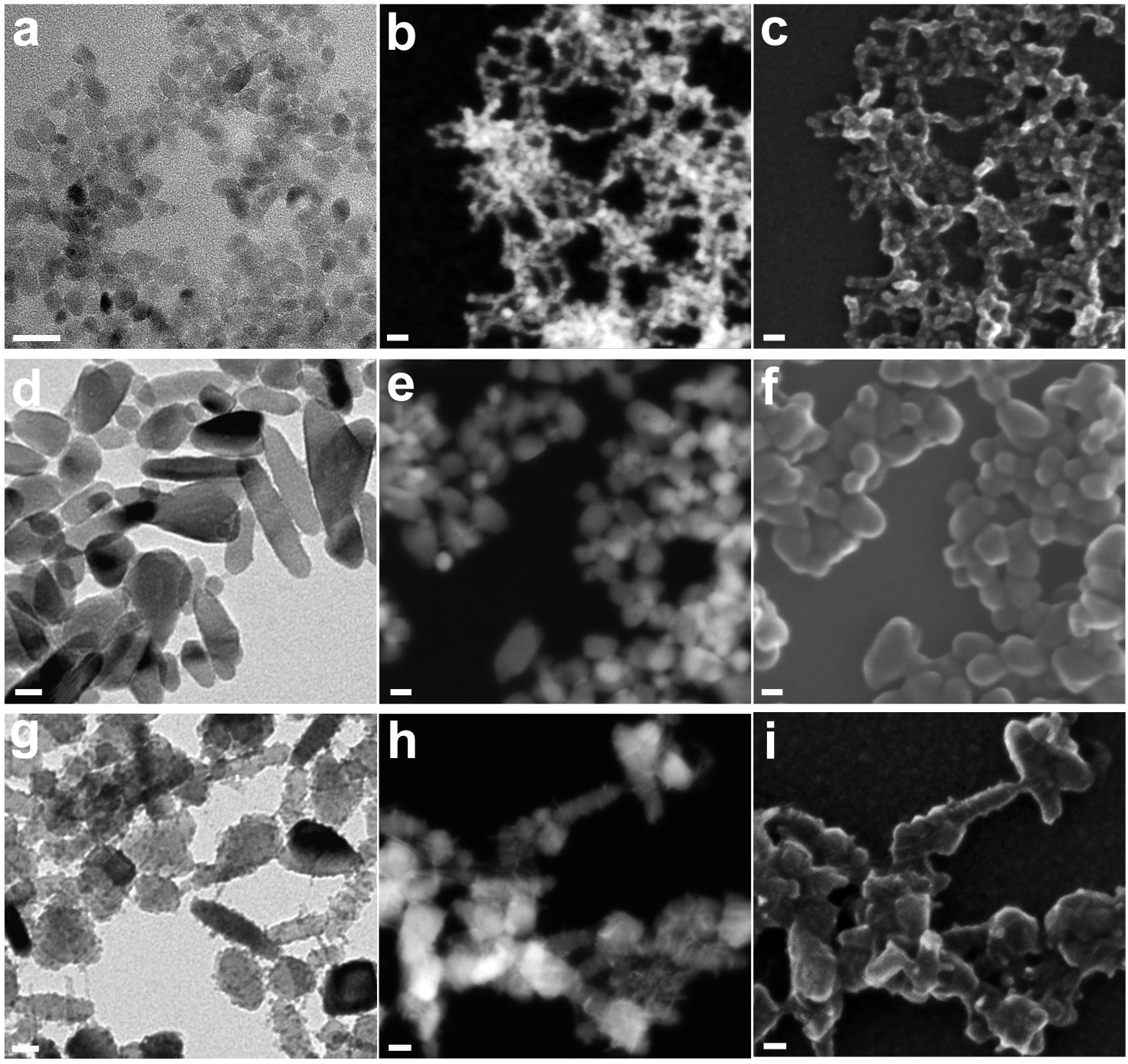


**Figure S1.** From left to right, low-resolution TEM, ADF-STEM in SEM, and SEM overviews of (a-c) W_x_Ti_1-x_O_2_; (d-f) starting TiO_2_ seeds; (g-i) TiO_2_-WO_3-x_. All scale bars are 20 nm.


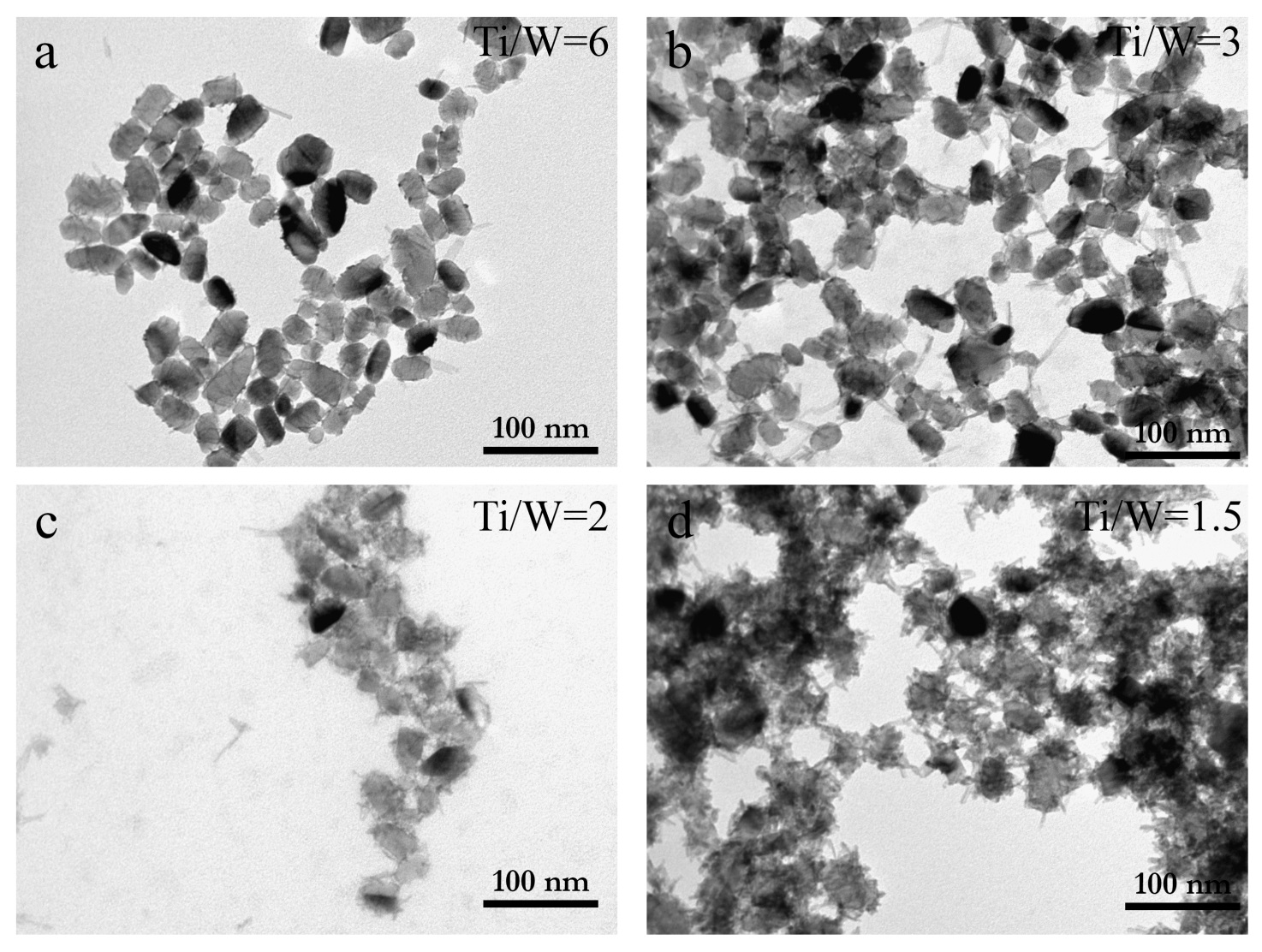


**Figure S2.** Representative TEM pictures of TiO_2_-WO_3-x_ heterostructures prepared at different Ti:W molar ratios. The Ti to W ratios reported over each image have been determined via ICP-AES analysis. Instead, the synthetic amounts of WCl_6_ injected within the same amount of TiO_2_ seeds (details in the experimental section) of each synthetic process are respectively: (a) 25 mg, (b) 50 mg, (c) 75 mg, (d) 100 mg in 8 ml anhydrous 2-propanol.


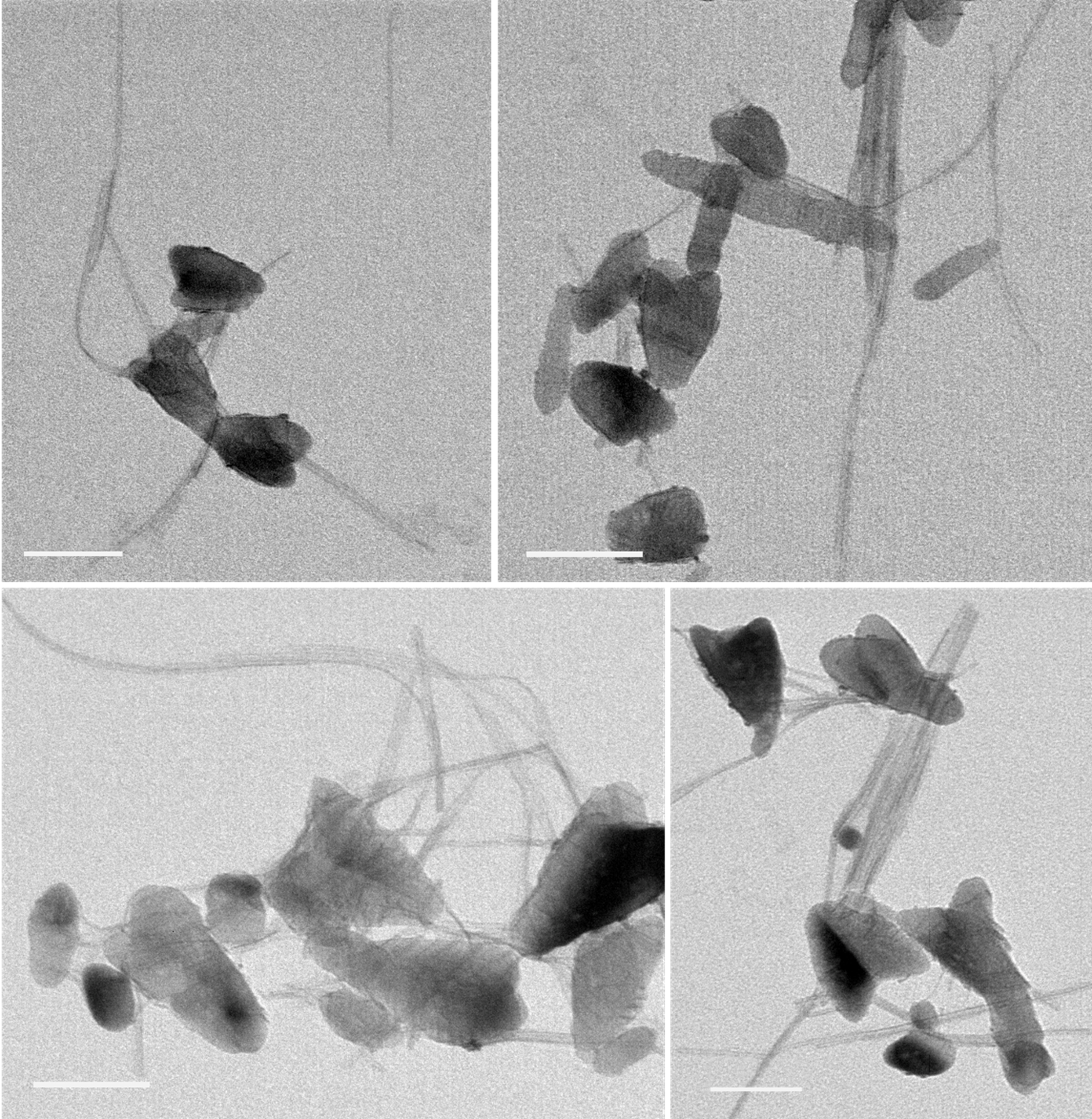


**Figure S3.** Representative TEM pictures of TiO_2_-WO_3-x_ heterostructures as obtained by scaling up 3 times a typical synthesis program as described in the experimental section. All scale bars are 50 nm.


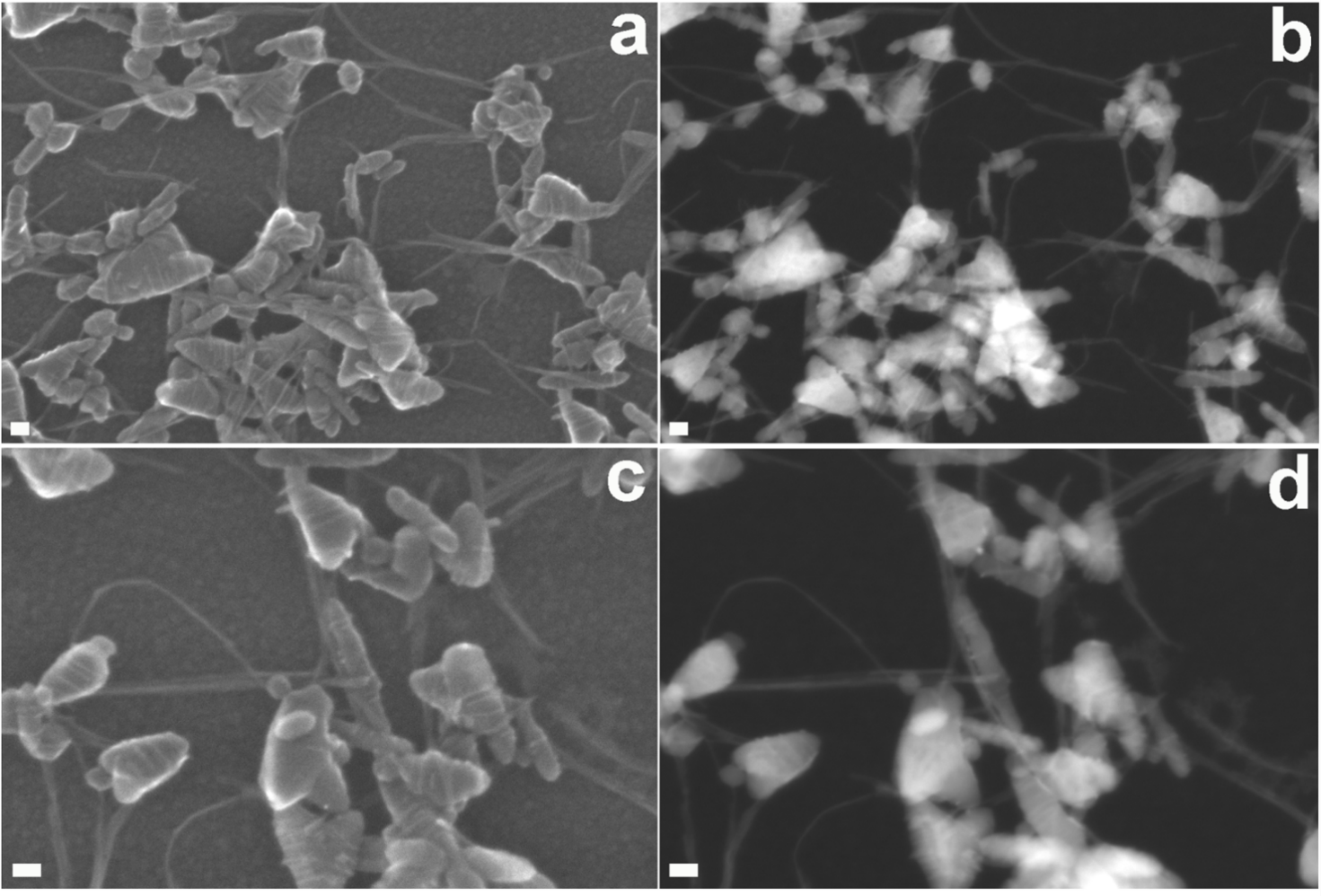


**Figure S4.** (a,c) SEM and (b,d) in-SEM ADF-STEM overviews, recorded on a TEM grid, of TiO_2_-WO_3-x_ heterostructures as obtained by tripling the amounts of the reagents of a typical synthesis recipe described in the experimental section. All scale bars are 20 nm.


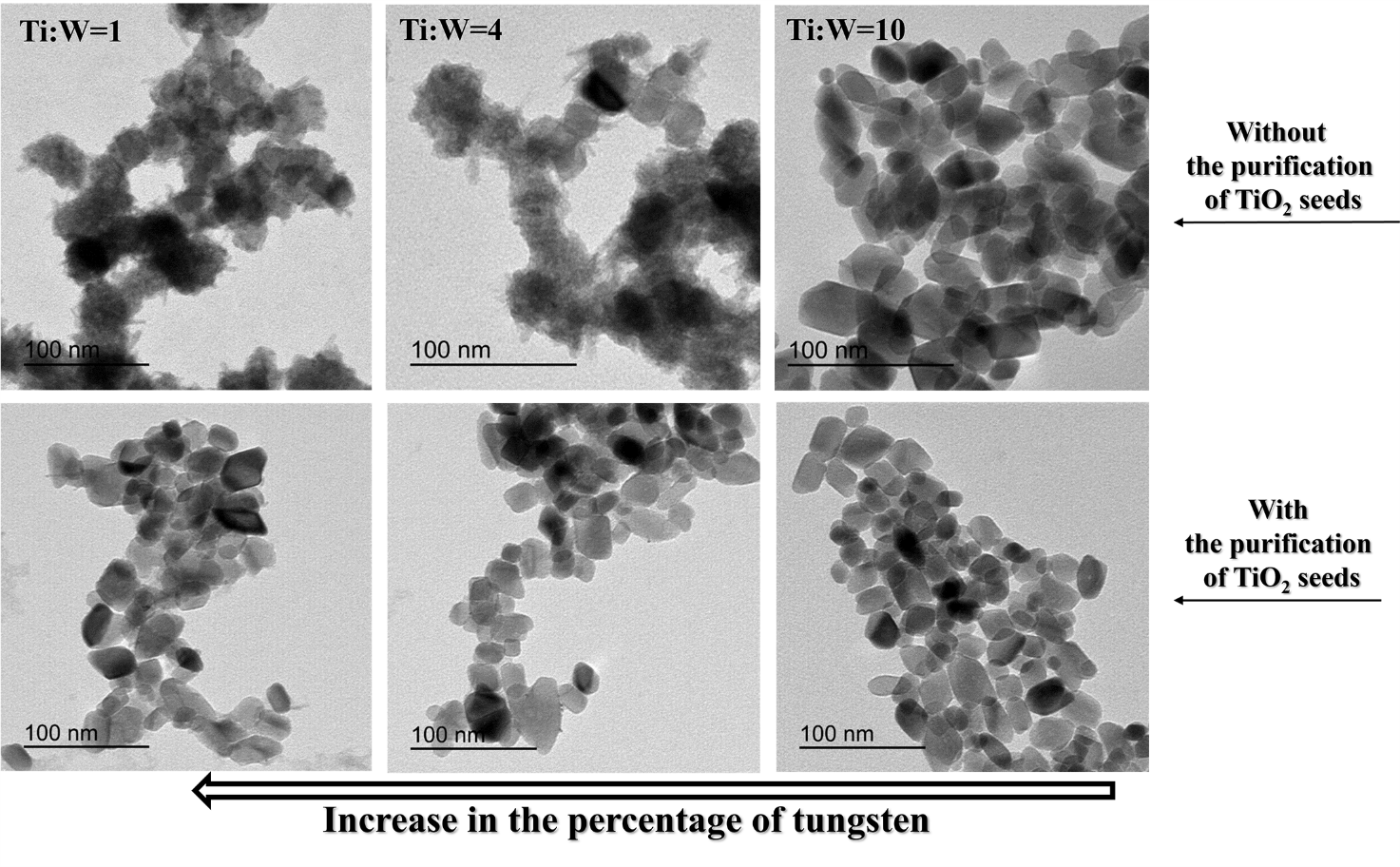


**Figure S5.** Effects upon hetero-structuring of Ti:W precursor molar ratio and of TiO_2_ seeds purification. Concerning the Ti-to-W molar ratios, the number of WO_3-x_ rod-like domains for each TiO_2_ seed resulted significantly dependent on the employed precursors’ molar ratios. Images report three different examples of heterostructures prepared following the same reaction conditions, however, at different Ti:W precursor molar ratios reported respectively in the image. Furthermore, the panel shows the effectiveness of tungsten precursors toward heterogeneous nucleation when occurring onto purified or unwashed TiO_2_ seeds.


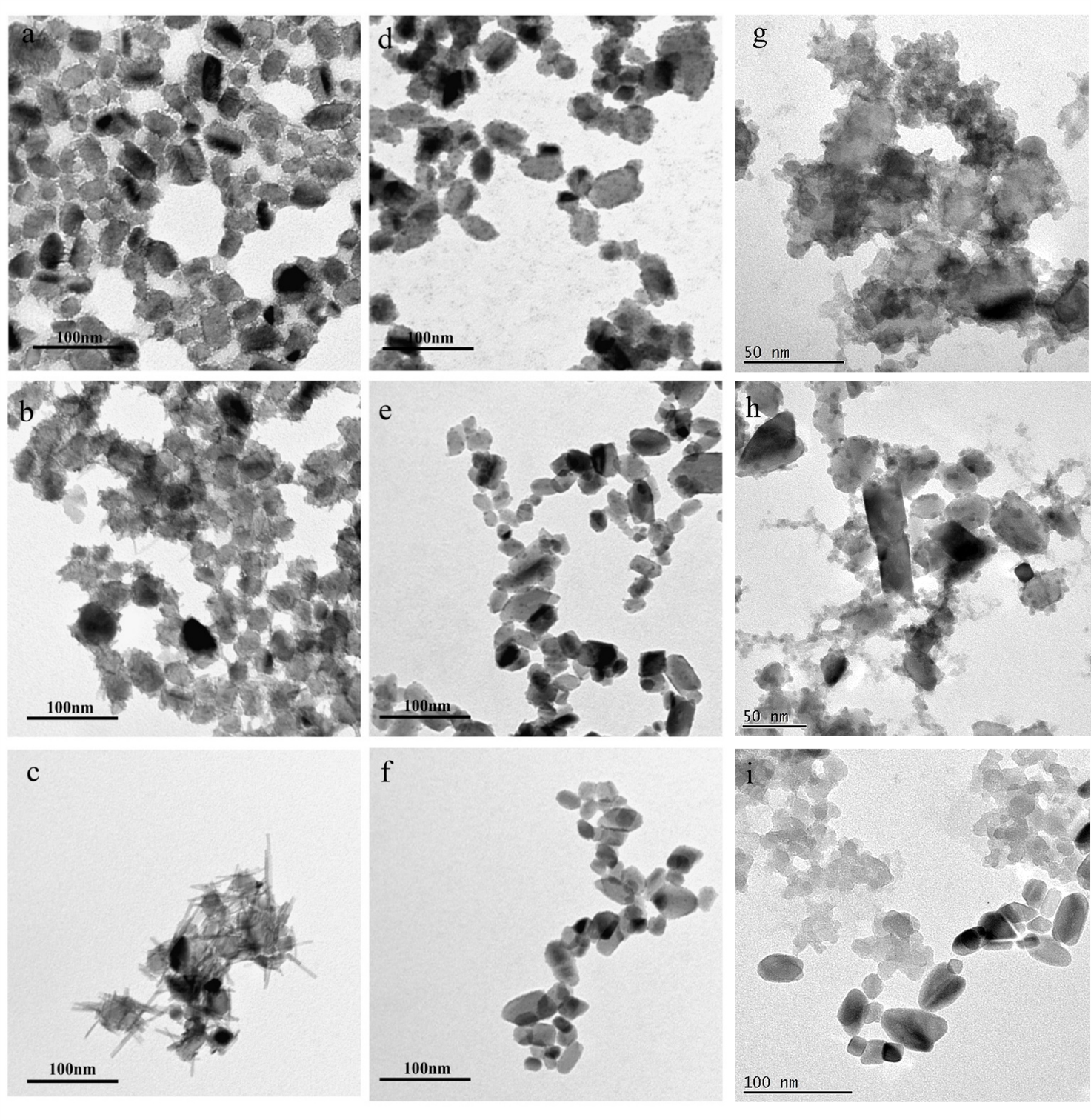


Figure S6. Comparison of heterostructured samples of TiO_2_-WO_3-x_, either obtained by the use of MW irradiation or conventional convective heating using a heating mantle or both approaches in sequence or autoclave; all the samples have been produced with a constant Ti:W precursor molar ratio of 4:1. (a) Sample refluxed at 100°C for 3h by heating mantle; (b) sample heated by MW irradiation at 100°C for 3h. Picture (c) shows TiO_2_-WO_3-x_ heterostructures obtained by further heating sample (a) at 180°C for 10 min through MW. Images (d-f) report samples developed at 180°C within an autoclave with Ti:W precursor molar ratio of 4:1 and (g-i) with constant Ti:W precursor molar ratio of 1:1, for a time growth of 30 min, 3 h, and 24 h, respectively.


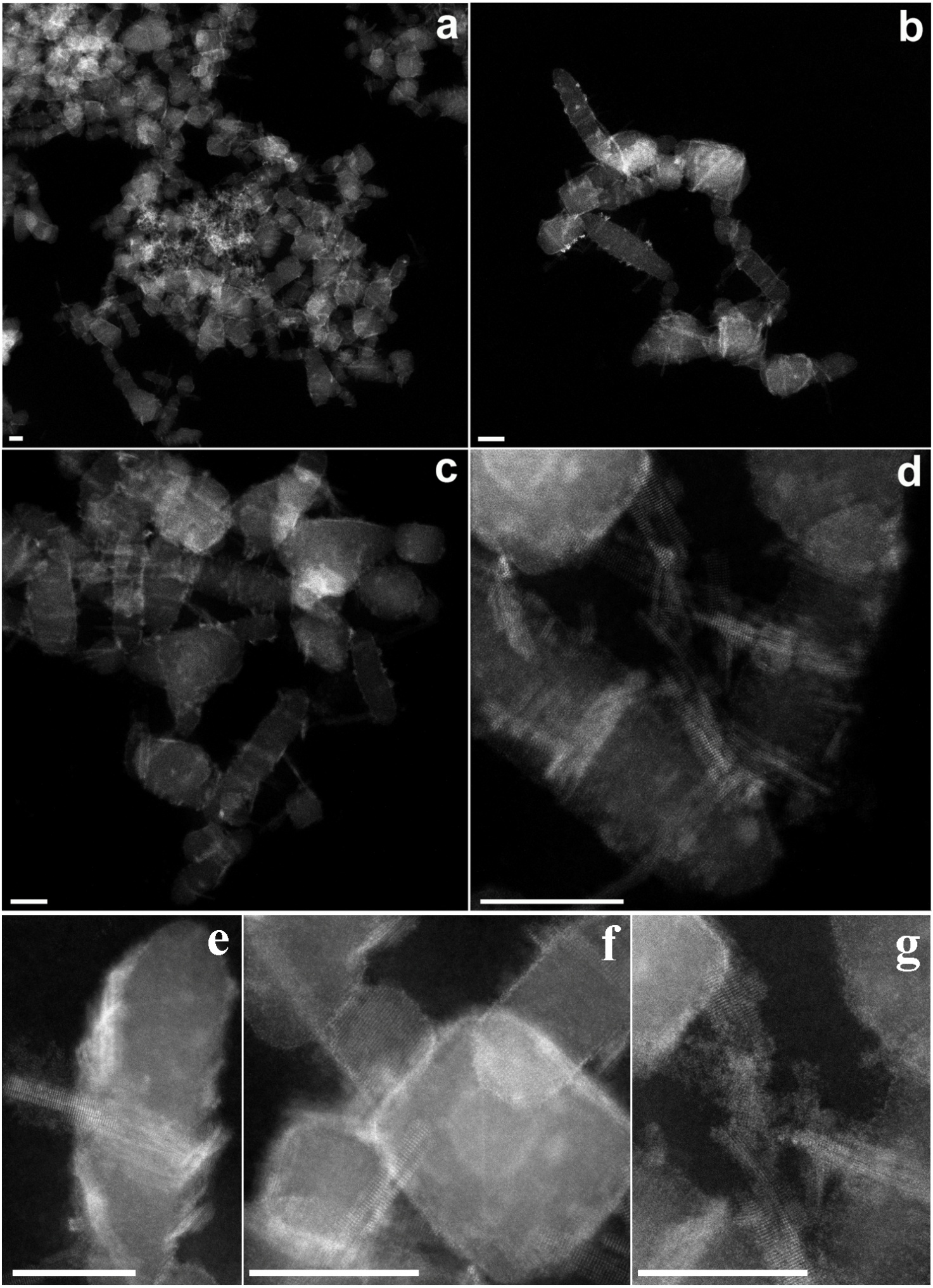


**Figure S7.** High-resolution ADF-STEM images of TiO_2_-WO_3-x_ nanocrystals. All scale bars are 20 nm.


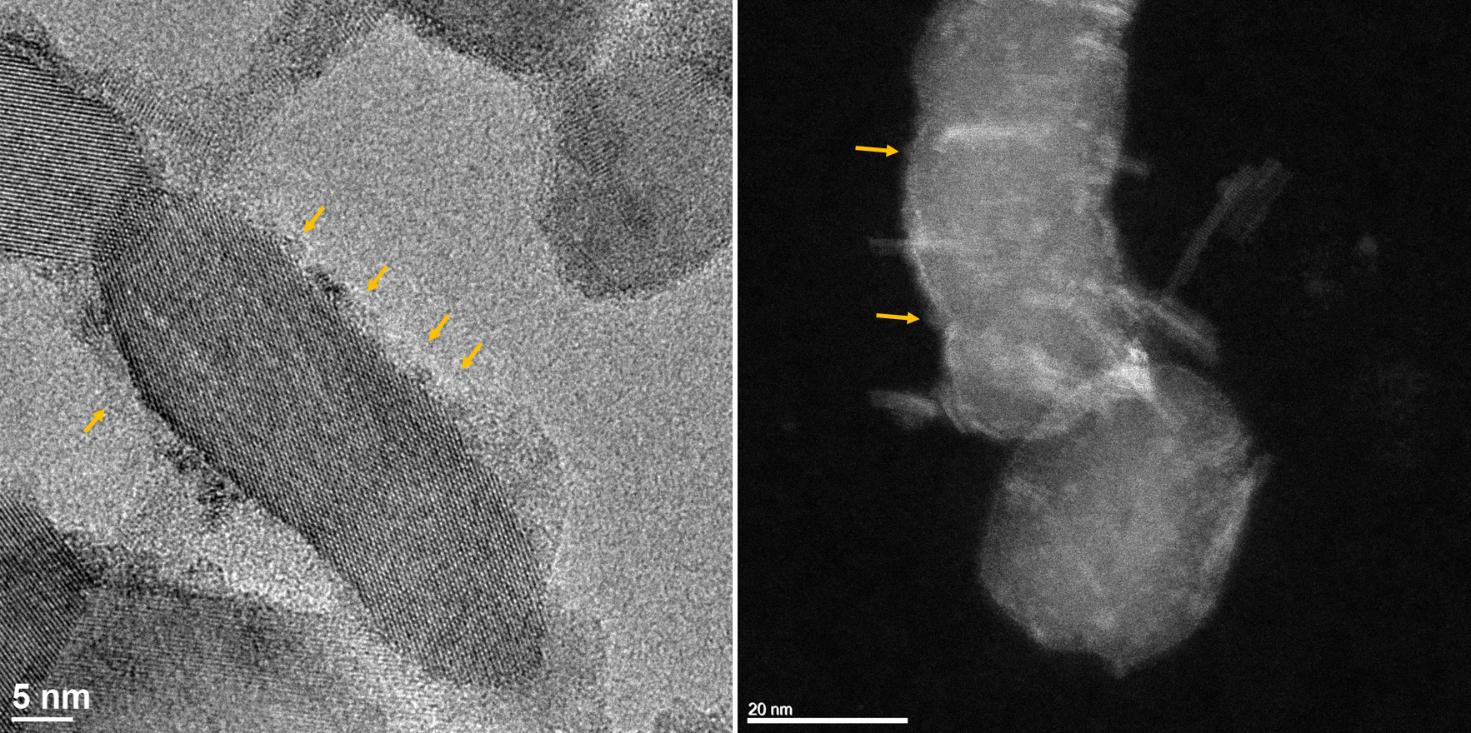


Figure S8. HRTEM and ADF-STEM of heterostructures, evidencing the existence of very few layers of tungsten oxide, plausibly amorphous, wrapping the TiO_2_ domain.


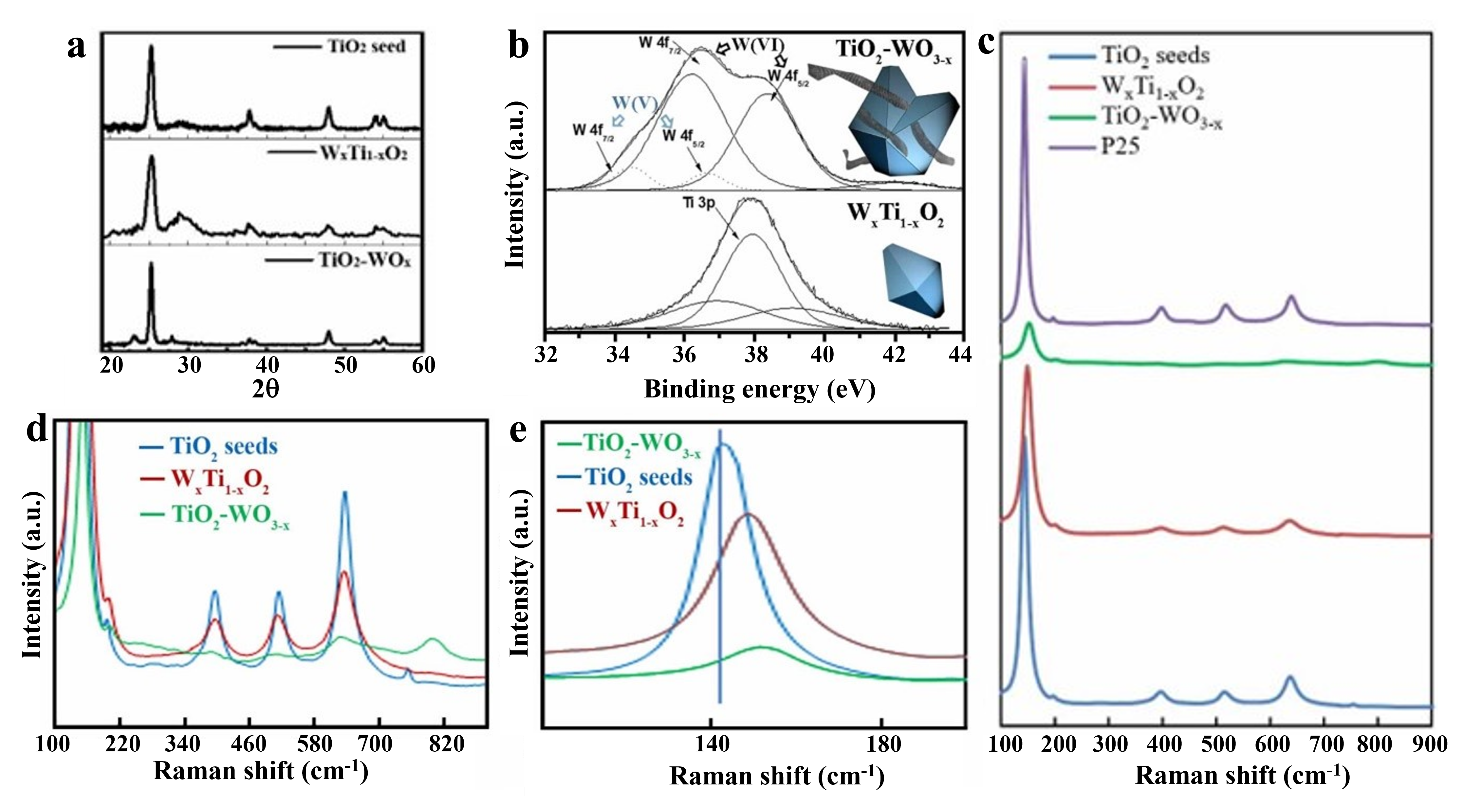


Figure S9. (a) XRD pattern of W_x_Ti_1-x_O_2_, TiO_2_-WO_3-x_, and TiO_2_ pristine seeds. (b) XPS of W_x_Ti_1-x_O_2_ and TiO_2_-WO_3-x_. (c-d) Complete Raman spectra of W_x_Ti_1-x_O_2_ and TiO_2_-WO_3-x_ NCs, TiO_2_ seeds, and TiO_2_ P25 commercial references for comparison. (e) 1-E_g_ zoom mode at 144.5 cm^-1^ of TiO_2_ seeds, W_x_Ti_1-x_O_2_ and TiO_2_-WO_3-x_ NCs and TiO_2_.

Structural and chemical characterization

Figure S9a reports the powder X-ray diffraction (XRD) patterns of W_x_Ti_1-x_O_2_, TiO_2_-WO_3-x,_ and TiO_2_ original seeds; reflections related to TiO_2_ anatase are distinguishable in bipyramidal-shaped ternary W_x_Ti_1-x_O_2_ oxide consistent with W-ions substitution in lieu of titanium with limited extent of doping (see Table S1 for quantitative ICP-AES analysis; W_x_Ti_1-x_O_2_ and TiO_2_-WO_3-x_ NCs have revealed a tungsten molar content of 2.5% and 39%, respectively).^[2]^

Figure S9b reports the XPS spectra of W_x_Ti_1-x_O_2_ and TiO_2_-WO_3-x_ NCs; all signals are summarized in Table S2. The Ti 2p spectrum of the samples is typical of the Ti^4+^ oxidation state as in TiO_2_, characterized by the main Ti 2p_3/2_ binding energy at around 459 eV (spectrum not shown).^[3]^ In the spectrum of TiO_2_-WO_3-x_ NCs, two doublets were fitted by applying constraints on the peak area ratio (W4f_5/2_/W4f_7/2_ = 0.75) and on the spin-orbit splitting ΔBE= (W4f_7/2_ -W4f_5/2_ ) = 2.15 eV. The doublet characterized by the 4f_7/2_ binding energy at 36.2 eV is attributed to W^6+^ from the WO_3-x_ nanobelts domain; the doublet with W 4f_7/2_ = 34.5 eV is attributable to W^5+^ as a consequence of the substoichiometric nature of WO_3-x_ nanobelts. Quantitative analyses provide 9% of W^5+^. A residual amount of nitrogen is observed in the case of TiO_2_ seeds, possibly derived from the synthetic path. The W 4f spectrum of W_x_Ti_1-x_O_2_ exhibits only one rather weak and broad doublet with W 4f_7/2_ binding energy at 36.7 eV ascribed to W^6+^. This peak suffers from the Ti 3p and W 4f binding energies overlapping.^[4]^ Because of the prominence of this peak, not recognizable in the heterostructured NCs, only a relatively limited amount of tungsten can be observed at the surface of W_x_Ti_1-x_O_2,_ unlike TiO_2_-WO_3-x_ NCs. The O 1s core level in pure TiO_2_ shows two components, one at 530.8 eV, which is attributed to the lattice oxygen, and another at higher binding energies referred to as surface hydroxyl groups (spectrum not shown). Based on comparison, both W_x_Ti_1-x_O_2_ and TiO_2_-WO_3-x_ higher binding energy components reveal a reduced contribution arising from hydroxyl groups, as a consequence of the tungsten-induced decrease of surface hydration,^[5]^ whereas a slightly negative energy shift occurs for the lower binding energy because of lattice rearrangement around oxygen bonding with heteroatom.^[3]^

Figure S9c-e reports a complete Raman analysis. The pristine TiO_2_ seeds (Figure S9c) exhibited the six characteristic peaks of the anatase phase at 144.5, 196, 396, 517, 636, and 750 cm^−1^ associated with 1-E_g_, 2-E_g_, B_1g_, A_1g,_ and 3-E_g_ modes of TiO_2_ anatase, respectively.^[6]^ As already highlighted in the main manuscript, a shift of the 1-E_g_ TiO_2_ main peak toward higher wavenumbers (Figure S9e) in relation to bare TiO_2_ seeds is observed in the presence of tungsten. The shift is attributed to the presence of oxygen vacancies in the crystal lattice as a follow-up of W-ions inclusion into the TiO_2_ crystal lattice^[3]^ and to the subsequent expansion of the anatase unit cell.^[7]^ These phenomena occurring in W-doped TiO_2_ anatase structures have been justified by ionic radii similarities existing between W^6+^ (0.600 Å) and Ti^4+^ (0.605 Å), whereas the lattice extension is attributed to the incorporation of W ions having oxidation states lower than +6 (0.660 Å for W^4+^ or 0.620 Å for W^5+^). Some anisotropic distortion of the anatase lattice can be induced by further different possible interactions with W cations and/or oxygen vacancies.^[5]^ The peak at ca. 806 cm^-1^ (Figure S9d) is the clearest signal of the monoclinic WO_3-x_.^[8]^ The other band of WO_3_ at 714 cm^-1^ is instead covered by that of anatase. The bands at 714 and 806 cm^-1^ correspond to the W–O–W stretching vibrations of the bridging oxygen atoms.^[9]^


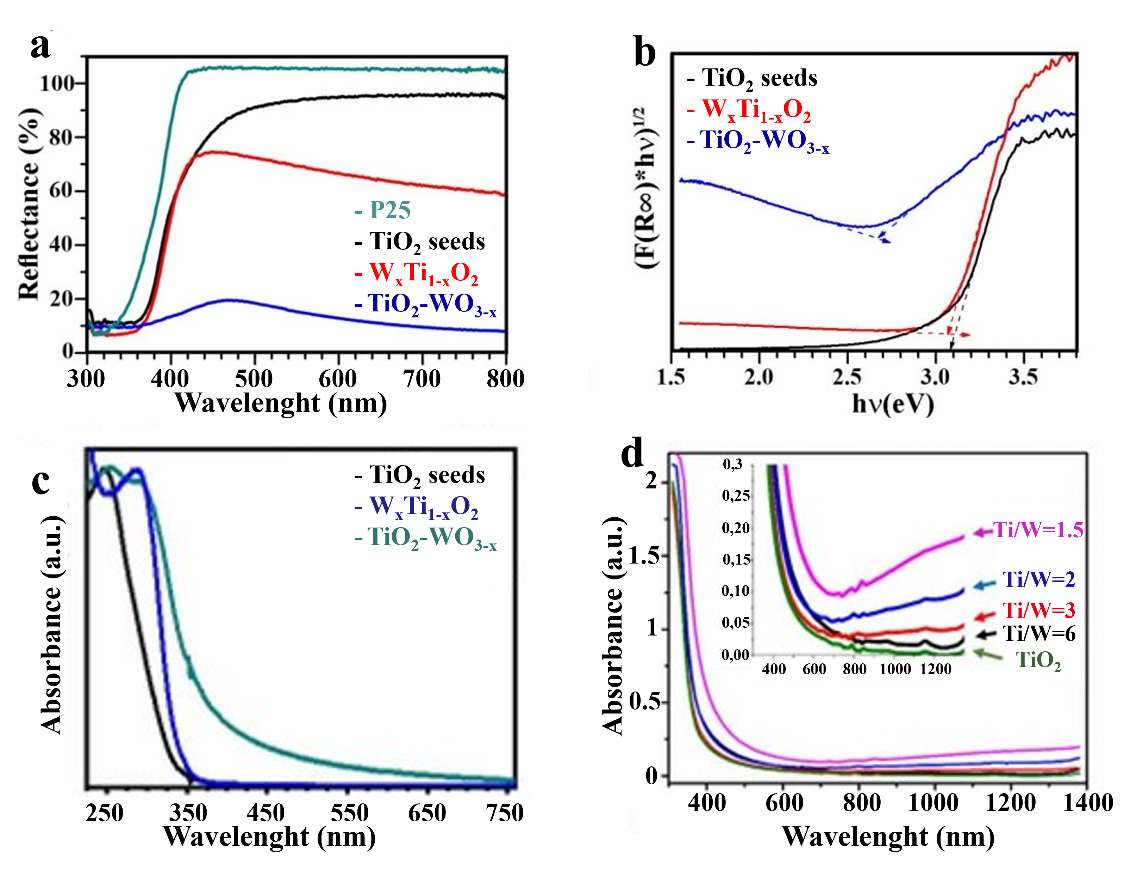


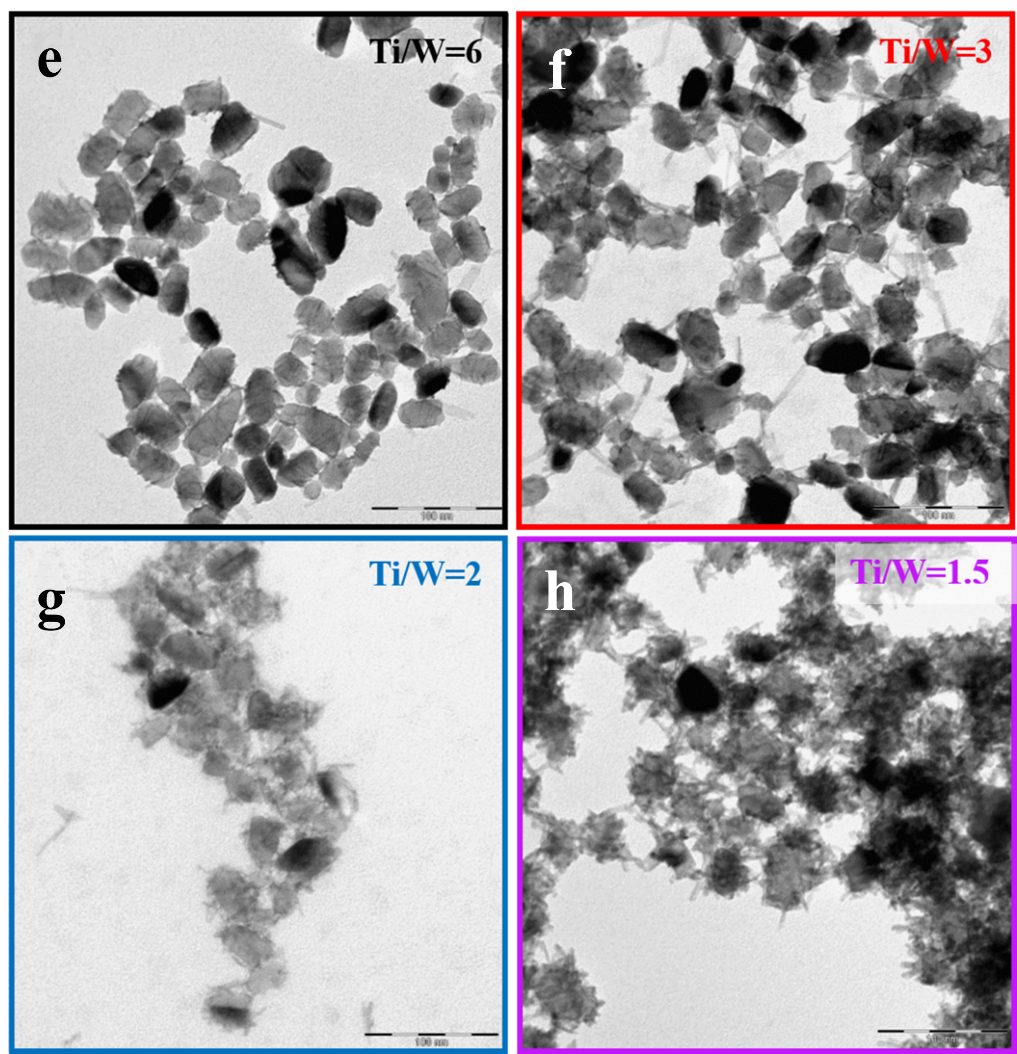


Figure S10. (a) DR spectra of MW-developed samples, namely TiO_2_ seeds, W_x_Ti_1-x_O_2,_ and TiO_2_-WO_3-x_ NCs, and commercial references. (b) Energy band gaps derived from the Kubelka-Munk treatment. (c) Extinction spectra of corresponding samples dispersed in aqueous media. (d) Extinction optical features in aqueous media and (e-h) corresponding TEM images of samples prepared at different Ti: W molar ratios; the same color code is adopted for optical spectra reported in picture (d) and TEM image frames.

Optical characterization

Figure S10c reports the absorbance spectra of samples dispersed in liquid media (water). Contrary to DRS investigations that are recorded by using a conspicuous amount of dried sample, those plots only very slightly evidence absorption increase in the wavelength range of Vis-NIR, because of in-solution sample dilution.

Figure S10d and Figure S10e-h correlate UV-Vis-NIR profiles and TEM images of heterostructures with gradual increasing of W precursor content; it appears clear that a critical reduction of the Ti: W molar ratio (W increase) is needed to distinctly detect LSPR in water-dispersed samples, although the overgrowth of WO_3-x_ nanobelts onto TiO_2_ seeds is well perceptible even for very low W content, as well. Indeed, clear evidence of vis-NIR (above 460nm) active LSPR of TiO_2_-WO_3-x_ is observed in dry powder (Figure S10a) and water-dispersed heterostructures enriched in W content (inset Figure S10d).

**Table S1.** W%-related content determined by ICP-AES analysis. Values of energy band gaps (last column) are also reported, obtained from the Kubelka-Munk function performed through optical analysis.

| Sample | Ti/W molar ratio | $\boldsymbol{W} \boldsymbol{mol}\boldsymbol{\%=}\frac{\mathbf{W}}{\mathbf{W+Ti}}\boldsymbol{\bullet100}$ | E_g_ (eV) |
| --- | --- | --- | --- |
| TiO_2_ seeds | 100 |  | 3.08 |
| W_x_Ti_1-x_O_2_ | 38.02 | 2.5% | 3.05 |
| TiO_2_-WO_3-x_ | 1.60 | 39% | 2.69 |
| TiO_2_ commercial (P25) |  |  | 3.18 |

**Table S2.** XPS data of developed W_x_Ti_1-x_O_2_ and TiO_2_-WO_3-x_ NCs and of pristine TiO_2_ seeds.

|  | Ti 2p_3/2_  (eV) | W 4f_7/2_  (eV) | O 1s  (eV) | N1s  (eV) | Ti at% | W at% | O at% | N  at% |
| --- | --- | --- | --- | --- | --- | --- | --- | --- |
| TiO_2_ seeds | 459.0 |  | 530.8(80%) (-O-)  532.2(20%) (-OH) | 403.8 | 28 |  | 64 | 8 |
| W_x_Ti_1-x_O_2_ | 459.0 | 36.7 (W^6+^) | 530.1(90%) (-O-)  532.0(10%) (-OH) |  | 31 | 2 | 67 |  |
| TiO_2_-WO_3-x_ | 458.6 | 36.2(91%) (W^6+^)  34.5(9%) (W^5+^) | 530.3(85%) (-O-)  531.9(15%) (-OH) |  | 12 | 24 | 64 |  |

**Figure S2.** Baseline approach to evaluate the bang gap energy from Tauc plot as reported by Macyk et. al.[1]


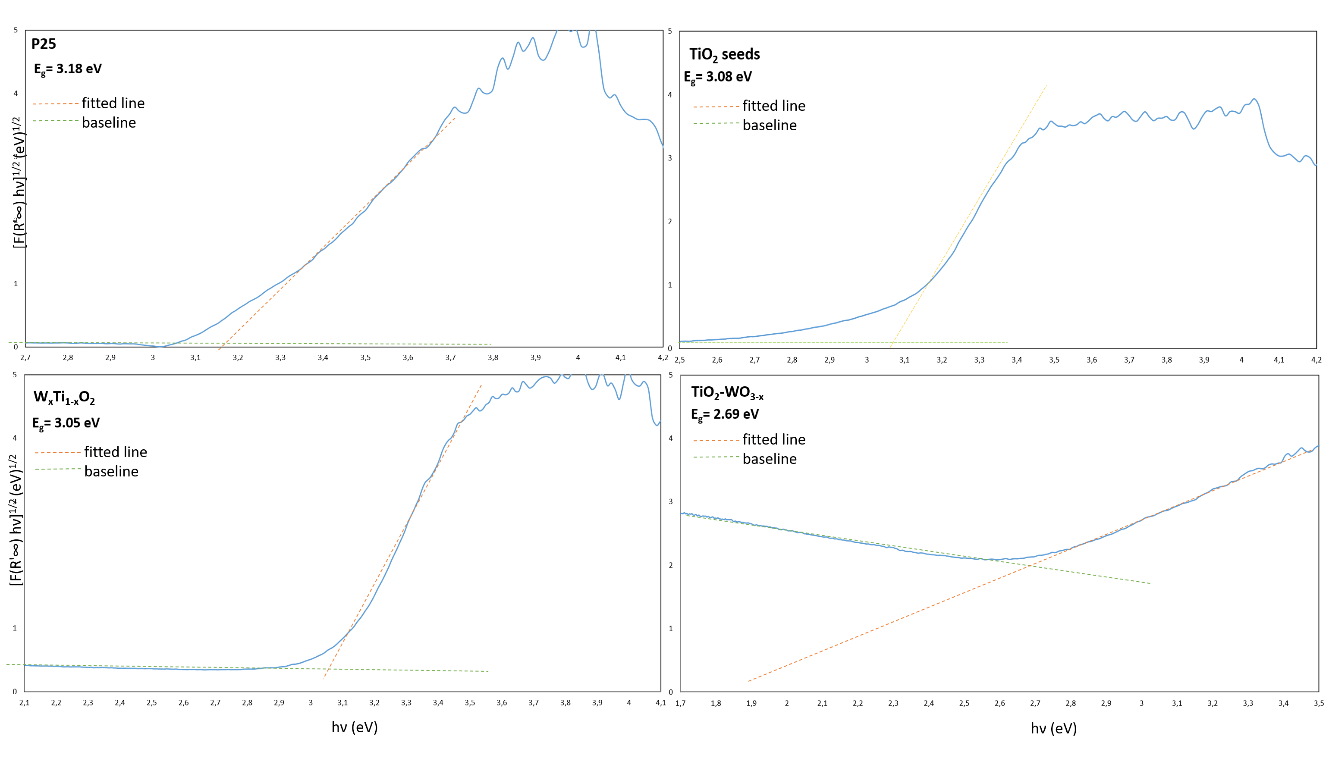


Figure S11. Baseline approach to evaluate the bandgap energy from the Tauc plot as reported by Macyk et. al.^[1]^.


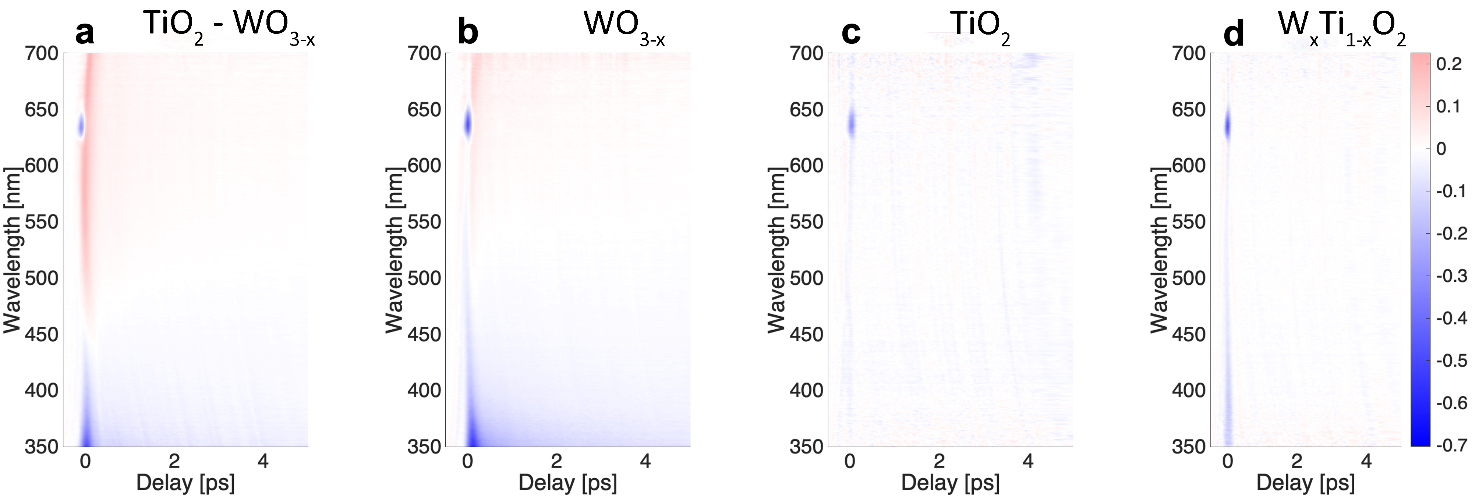


**Figure S12.** Pump-probe transient reflectivity maps exciting the samples in the near-infrared (800nm) for different systems: (a) TiO_2_-WO_3-x_, (b)WO_3-x_, (c) TiO_2_, (d) Ternary (W_x_Ti_1-x_O_2_). Only TiO_2_-WO_3-x_ and WO_3-x_ show a negative-positive signal persistent at longer times compared to the pulse duration, attributed to the plasmonic response. The strong and ultrashort negative feature present in all the maps at about 650 nm is attributed to coherent Raman scattering of the water solvent.


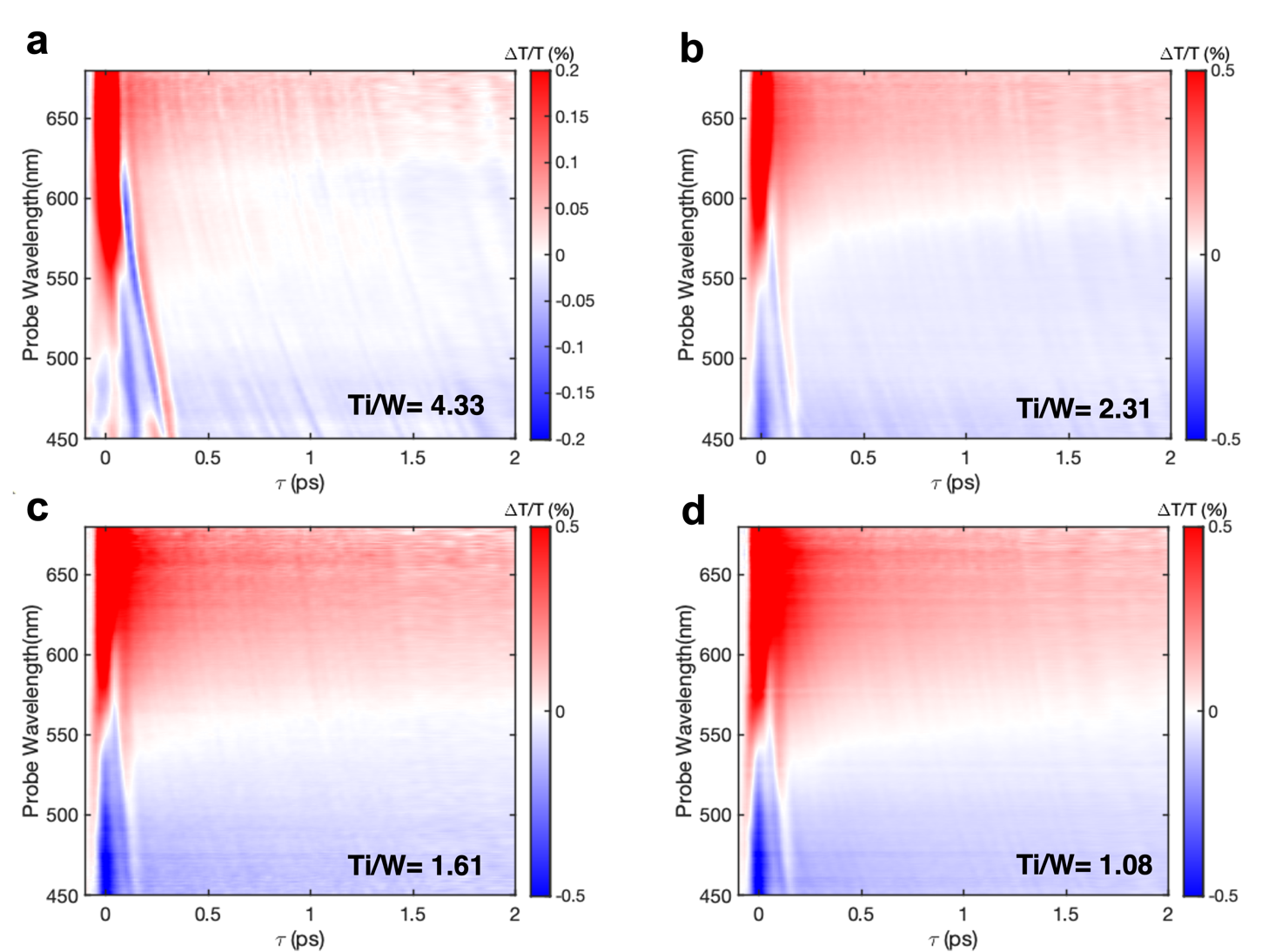


Figure S13. (a-d) Pump-probe transient transmission maps exciting the plasmon resonance of the heterostructure with NIR pulses tuned at 1000 nm, compressed to < 20 fs for TiO_2_-WO_3-x_ heterostructures with a Ti/W content ratio of 4.33 (a), 2.31 (b), 1.61 (c), 1.08 (d), respectively.

**Table S3.** Liquid-phase 4-MBA partial oxidation results. X, S, and Y, respectively, represent conversion, selectivity, and yield after 4 h of UV irradiation. The corresponding QE values are listed in the final column.

| SOLID CATALYST | X_t=4h_  $\left[ \frac{\text{C}_{\text{0}}\text{-}\text{C}_{\text{r}}}{\text{C}_{\text{0}}} \right]\text{×}\text{100}$ | S_AA t=4h_  $\left[ \frac{\text{C}_{\text{p}}}{\text{C}_{\text{0}}\text{-}\text{C}_{\text{r}}} \right]\text{×}\text{100}$ | Y_AA t=4h_  $\left[ \frac{\text{C}_{\text{p}}}{\text{C}_{\text{0}}} \right]\text{×}\text{100}$ | S_Acid_  $\left[ \frac{\text{C}_{\text{p}}}{\text{C}_{\text{0}}\text{-}\text{C}_{\text{r}}} \right]\text{×}\text{100}$ | S_AA_ _x=30_  $\left[ \frac{\text{C}_{\text{p}}}{\text{0.7C}_{\text{0}}} \right]\text{×}\text{100}$ | t_x=30_  (min) | QE (%) |
| --- | --- | --- | --- | --- | --- | --- | --- |
| UV irradiation | | | | | | |  |
| TiO_2_ seeds | 55 | 32 | 18 | 4 | 45 | 120 | 0.045 |
| W_x_Ti_1-x_O_2_ | 50 | 42 | 21 | 4 | 55 | 135 | 0.075 |
| TiO_2_-WO_3-x_ | 60 | 34 | 21 | 14 | 68 | 90 | 0.070 |
| TiO_2_ commercial (P25) | 52 | 20 | 11 | 1 | 16 | 75 | 0.060 |


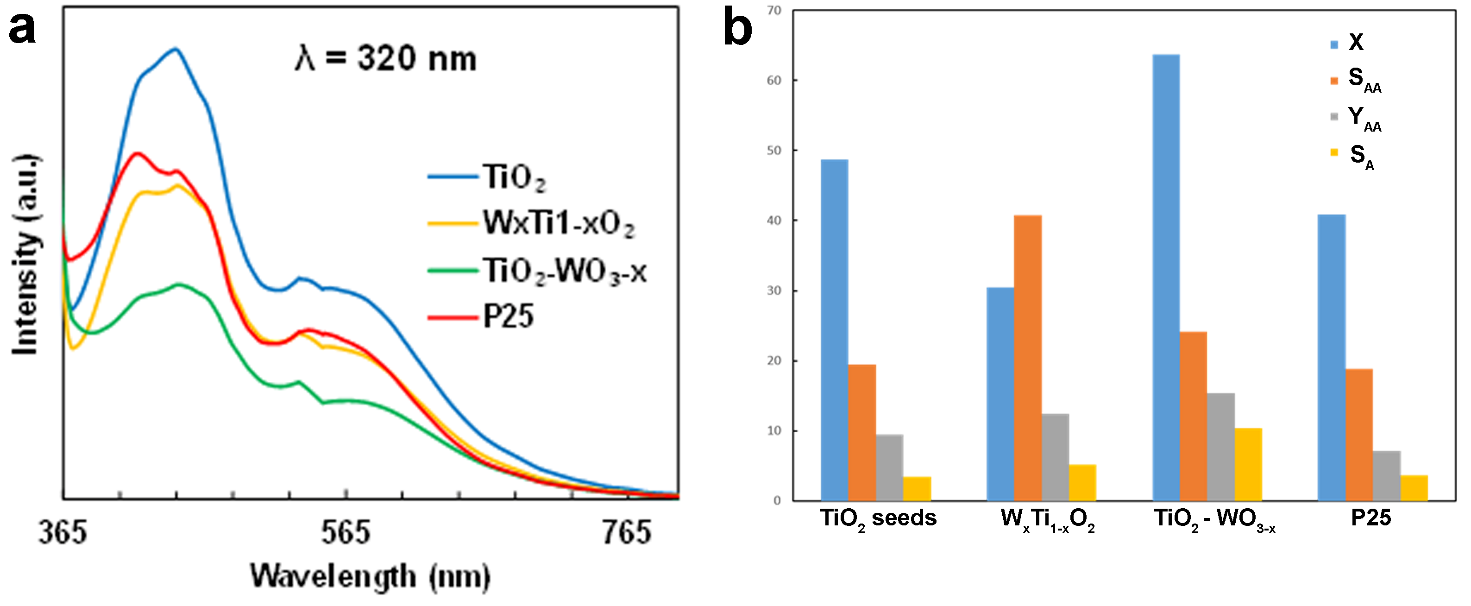


Figure S14. (a) Photoluminescence spectra of the different samples (λ_excitation_ = 320 nm). Two main emission features can be observed: the peak at ca. 420 nm is related to the band-to-band emission of TiO_2,_ while the one at ca. 440 nm is attributed to the self-trapped excitons localized on the TiO_6_ octahedral.^[10]^ The peaks at higher wavelength are ascribed to electrons trapped in the defect centres connected with surface oxygen vacancies. (b) 4-MBA conversion, selectivity, and yield to the corresponding aldehyde and acid after 4 h of simulated solar light irradiation.

Quantum efficiency (QE) determination^[11]^

The energetic efficiency of the heterogeneous photocatalytic system can be determined by evaluating two quantities: (i) the rate of photon absorption (*rpa*) and (ii) the specific reaction rate (*srr*):

$\boldsymbol{rpa=}\frac{\text{number of absorbed photons}}{\text{time}\boldsymbol{\cdot}\text{surface area}} \left[ \frac{\boldsymbol{mol}}{\boldsymbol{s\cdot}\boldsymbol{m}^{\boldsymbol{2}}} \right]$ (1)

$\boldsymbol{srr=}\frac{\text{number of reacted molecules}}{\text{time}\boldsymbol{\cdot}\text{surface area}} \left[ \frac{\boldsymbol{mol}}{\boldsymbol{s\cdot}\boldsymbol{m}^{\boldsymbol{2}}} \right]$ (2)

Both of these rates should be evaluated based on the active sites, but this last quantity is very difficult to determine experimentally, so the BET surface area can be used as an approximate reference parameter. From the experimental determination of *rpa* and *srr*, the quantum efficiency can be found and used to compare different photocatalysts:

$\boldsymbol{\eta}\left( \boldsymbol{\lambda} \right)\boldsymbol{=}\frac{\boldsymbol{number of reacted molecules}}{\boldsymbol{number of photons absorbed}}$ (3)

The number of absorbed photons was determined by neglecting the diffusion and/or reflection phenomena and considering only the photons emitted by the lamp (*φ_i_*) and the photons transmitted by the dispersion (*φ_t_*). Then the absorbed photons (*φ_a_*) can be calculated as follows:

$\boldsymbol{\varphi}_{\boldsymbol{a}}\boldsymbol{=}\boldsymbol{\varphi}_{\boldsymbol{i}}\boldsymbol{-}\boldsymbol{\varphi}_{\boldsymbol{t}}$ (4)

To maximize the exploitation of the photons emitted by the lamp, the optimum amount of photocatalyst was determined by adding quantities of the powder to the reacting solution and measuring the transmitted flow at the external wall of the reactor. When the transmitted flow reached 10% of the emitted flow, we selected the corresponding weight of the solid. A non-zero value of the transmitted flow ensured that all the catalyst was adequately irradiated, thereby avoiding any shielding effects.

*φ_i_* was measured by filling the reactor with water and measuring, using a radiometer, its value at the external wall of the reactor. As reported in the main manuscript, a quartz halogen and a pure UV lamp were used. See details in the experimental section of the main manuscript.

A photocell was used for the measurements of the emitted and transmitted flows, in the range 315 – 400 nm.

φ_a_ is determined according to equation (4), hence, considering that φ_t_ is the 10% of φ_i_, for the employed lamps we have the following values

$\boldsymbol{\varphi}_{\boldsymbol{i UV}}\boldsymbol{=100}\frac{\boldsymbol{W}}{\boldsymbol{m}^{\boldsymbol{2}}}$ and $\boldsymbol{\varphi}_{\boldsymbol{t UV}}\boldsymbol{=10}\frac{\boldsymbol{W}}{\boldsymbol{m}^{\boldsymbol{2}}}$ $\boldsymbol{\varphi}_{\boldsymbol{a UV}}\boldsymbol{=90}\frac{\boldsymbol{W}}{\boldsymbol{m}^{\boldsymbol{2}}}$

$\boldsymbol{\varphi}_{\boldsymbol{i halogen}}\boldsymbol{=38}\frac{\boldsymbol{W}}{\boldsymbol{m}^{\boldsymbol{2}}}$ and $\boldsymbol{\varphi}_{\boldsymbol{t halogen}}\boldsymbol{=3.8}\frac{\boldsymbol{W}}{\boldsymbol{m}^{\boldsymbol{2}}}$ $\boldsymbol{\varphi}_{\boldsymbol{a halogen}}\boldsymbol{=34.2}\frac{\boldsymbol{W}}{\boldsymbol{m}^{\boldsymbol{2}}}$

The energy of a photon at a certain wavelength λ is:

$\boldsymbol{E}\left( \boldsymbol{\lambda} \right)\boldsymbol{=}\frac{\boldsymbol{h}\left[ \boldsymbol{J}\boldsymbol{\cdot}\boldsymbol{s} \right]\boldsymbol{\cdot}\boldsymbol{c}\left[ \frac{\boldsymbol{m}}{\boldsymbol{s}} \right]}{\boldsymbol{\lambda}\left[ \boldsymbol{m} \right]}$ (5)

in which h is the Planck constant and c the light speed. For a mole of photons (1 Einstein), it should be multiplied by the Avogadro number (N_A_).

By considering the average values of the two wavelengths (*i.e.,* λ = 357.5 nm), the following energy of the photons was calculated by means of equation (5):

E(λ) = $\frac{\boldsymbol{h\cdot}\boldsymbol{c}}{\boldsymbol{\lambda}}\boldsymbol{=}\frac{\boldsymbol{6.626\cdot}\boldsymbol{10}^{\boldsymbol{-34}} \left[ \boldsymbol{J}\boldsymbol{\cdot}\boldsymbol{s} \right]\boldsymbol{\cdot3\cdot}\boldsymbol{10}^{\boldsymbol{+8}}\boldsymbol{[}\frac{\boldsymbol{m}}{\boldsymbol{s}}\boldsymbol{]}}{\boldsymbol{357.5\cdot}\boldsymbol{10}^{\boldsymbol{-9}}\boldsymbol{[}\boldsymbol{m}\boldsymbol{]}}\boldsymbol{=5.56\cdot}\boldsymbol{10}^{\boldsymbol{-19}}\boldsymbol{[}\boldsymbol{J}\boldsymbol{]}$

This corresponds to the following energy for 1 mol of photons:

*rpa* (λ) = moles of photons absorbed (λ) =$\frac{\boldsymbol{\varphi}_{\boldsymbol{a}}}{\boldsymbol{E}\left( \boldsymbol{\lambda} \right)\boldsymbol{\cdot}\boldsymbol{N}_{\boldsymbol{A}}}\boldsymbol{=}\frac{\boldsymbol{\varphi}_{\boldsymbol{a}}\boldsymbol{[}\frac{\boldsymbol{J}}{\boldsymbol{s\cdot}\boldsymbol{m}^{\boldsymbol{2}}}\boldsymbol{]}}{\boldsymbol{E}\left( \boldsymbol{\lambda} \right) \left[ \boldsymbol{J} \right]\boldsymbol{\cdot}\boldsymbol{N}_{\boldsymbol{A}}\boldsymbol{[}\frac{\boldsymbol{1}}{\boldsymbol{mol}}\boldsymbol{]}}$

The specific reaction rate (*srr*) is given by:

$\boldsymbol{ssr=-r=}\frac{\boldsymbol{1}}{\boldsymbol{S}}\frac{\boldsymbol{dN}}{\boldsymbol{dt}}\boldsymbol{=}\frac{\boldsymbol{V}}{\boldsymbol{S}}\frac{\boldsymbol{dC}}{\boldsymbol{dt}}\boldsymbol{=k}$ (the reaction order is zero)

Where V is the volume of the reacting solution. S is the surface area of the photocatalyst (BET surface area), C is the concentration of the reacted molecules (reactants), and k is the reaction rate constant (since the reaction order is zero). The rate of change of concentration is:

$\frac{\boldsymbol{dC}}{\boldsymbol{dt}}\boldsymbol{=}\frac{\boldsymbol{S}}{\boldsymbol{V}}\boldsymbol{k}$

$\boldsymbol{C=}\boldsymbol{C}_{\boldsymbol{0}}\boldsymbol{-}\frac{\boldsymbol{S}}{\boldsymbol{V}}\boldsymbol{kt}$

The values of k were determined by applying a least-squares best-fitting procedure to the experimental data.

Nanostructure growth mechanisms

Hereafter, a synthetic rationalization of the growth mechanisms of titanium- and tungsten-oxide-based nanostructures is suggested. Concerning the first growth strategy, which provides for the co-injection of both metal precursors, in either their liquid form and in a corresponding alcoholic solution, metal halide precursors are made to react with a reagent, based on the same or different metal, in the alkoxide form; these latter mostly result in less reactive than halides. This rationale has been followed in several experimental examples, where chloride-based metallic reagents (MCl_x_) have been mixed with isopropoxides (M(OPr^i^)_x_) with the aim at increasing the reaction kinetics.^[12]^

In the experimental cases investigated herein, an anhydrous isopropanol-based solution of WCl_6_ is employed as the original reagent of tungsten. As we know, under non-acidic conditions, the formation of an alcoholic mixture of WCl_6-x_(OPr^i^)_x_ and WCl_5-x_(OPr^i^)_x_ is expected, confirmed by the blue color of the solution.^[13]^ When mixed with titanium isopropoxide, the formation of intermixed-metal chloroisopropoxide species can promote different chemical precursor condensation paths during microwave heating. Further, the alkaline ambient due to tetramethylammonium hydroxide accelerates the process of intermetallic complexes formation, promoted by the TiO^-^ nucleophilic species, which undergo condensation reactions.^[14]^ Intermetallic chloroisopropoxides reaction intermediates formed by mixing M^1^(OPr^i^)_x_ with a solution of M^2^Cl_y_ have been demonstrated.^[15]^ It’s worth saying that the supposed reaction will realistically compete with ligand redistribution reaction (ligand exchange) between metal chlorides and metal alkoxides that can occur at room temperature, leading to metal chloroisopropoxide compounds with several molar ratios, described as the overall formula M(O^i^Pr)_x_Cl_4-x_ (M=metal; with 0.5<x<4). All these pieces of evidence corroborate the hypothesis based on the formation of intermixed M^1^M^2^chloroisopropoxide molecular complexes as chemical intermediates afterward involved in the events of nucleation; presumably, the kinetic rates of hetero- and homo-condensation reactions compete proportionately, depending on the original amounts of reagents.

Concerning the development of TiO_2_-WO_3-x_ heterostructures, the experimental proofs suggest that: *i)* the thermodynamically favorable heterogeneous nucleation of a new phase onto the surface of preformed seeds is the most evident mechanism according to which the growth occurs. The interphase lattice mismatch leads the growth, promoting, at first, the formation of an even sub-nm coverage of tungsten oxide from which elongated structures of the same material branch out. Such protruding structures appear 2D stripe-like, and thin enough that, in some images, they coil the original seed or fold up on it. Even the usage of MW does not endorse the homogeneous evolution of the pure WO_3-x_ phase.

*ii)* Original TiO_2_ seeds dispersed in water undergo a superficial hydroxylation thus exposing –OH moieties, therefore, the heteronucleation of the tungsten precursor takes place via nucleophilic attack promoted by the superficial –OH groups and is catalyzed by TMAH residual, the presence of which is mandatory in solution (see Figure S5 and Ref.^[16]^).

*iii)* Hydroxyl groups, and possibly interacting tetramethylammonium cations, spread over the seed surface, dramatically enhance the selectivity of the surface toward MW over-heating, thus contributing to catalyzing paths of reaction unavailable by other conventional procedures (refer to all images in Figure S6) under these experimental conditions; no nanostructures are nucleated out of the surface. TiO_2_ seeds operate as a MW absorbing material through a dielectric loss mechanism and more specifically through an interface polarization loss because of –OH groups grafted onto the surface that behave as polarization sites.^[17]^ In that view, also charges built up at the surface create a loss mechanism of interfacial polarization. Therefore, the surface is designed as a specific substrate for accommodating MW overheating.

*iv)* Finally, as soon as the growth of the heterostructures is ignited, MW-assisted seed surface activation is not the only expectation of growth; the high temperatures readily accessible in MW reactors, and therefore the prompt availability (reactivity) of WCl_6_ in its original or otherwise complexed form, represent the most critical factors driving the growth under kinetic control (Figure S6a-c). WCl_6_ generates nuclei onto the seed surface that grow as long belt-like shapes of WO_3-x_, favored by its MW sensitivity and the quickly achieved high-temperature conditions. Heterointerface engineering of the seed surface because of the appearance of a sub-nm W-based thin and discontinuous shell (Figure 2d-f and Figure S8) may represent an additional contribution to MW attenuation (dielectric loss due to dipole polarization) and therefore another form of activation of the seed surface towards MW.^[18]^ It is also worth noting that upon increasing the WCl_6_-to-TiO_2_ seeds molar ratio in the reaction mixture, no significant variation in the nanobelt length has been observed; however, more nuclei form on the heterosurface, thereby promoting the formation of denser regions of branching protruding out of the seed (Figure S2). This last point is reflected in the literature, although it is related to a different reaction ambient and approach, and to micrometer-sized systems.^[19]^ Excessive additions of WCl_6_, rather, promote kinetic regimes of growth that can sustain homogeneous nucleation of isolated WO_3-x_ nanocrystals as well as non-isotropic development.

_______________________________________________________________________________________

References

[1] P. Makuła, M. Pacia, W. Macyk, *J. Phys. Chem. Lett.* 2018, *9*, 6814.

[2] J. Li, J. Xu, W.-L. Dai, H. Li, K. Fan, *Appl. Catal. B-Environ.* 2008, *82*, 233.

[3] Y. Li, A. G. Walsh, D. Li, D. Do, H. Ma, C. Wang, P. Zhang, X. Zhang, *Nanoscale* 2020, *12*, 17245.

[4] F. Chang, J. Sun, J. Wang, X. Wang, B. Deng, X. Hu, *Colloids and Surfaces A: Physicochemical and Engineering Aspects* 2016, *511*, 329.

[5] F. Riboni, L. G. Bettini, D. W. Bahnemann, E. Selli, *Catal. Today* 2013, *209*, 28.

[6] T. Ohsaka, F. Izumi, Y. Fujiki, *J. Raman Spectrosc.* 1978, *7*, 321.

[7] a) S. Sathasivam, D. S. Bhachu, Y. Lu, N. Chadwick, S. A. Althabaiti, A. O. Alyoubi, S. N. Basahel, C. J. Carmalt, I. P. Parkin, *Sci. Rep.* 2015, *5*, 10952; b) B. Santara, B. Pal, P. K. Giri, *J. Appl. Phys.* 2011, *110*, 114322.

[8] a) H. Zheng, J. Z. Ou, M. S. Strano, R. B. Kaner, A. Mitchell, K. Kalantar-zadeh, *Adv. Funct. Mater.* 2011, *21*, 2175; b) A. Di Paola, M. Bellardita, B. Megna, F. Parrino, L. Palmisano, *Catal. Today* 2015, *252*, 195; c) G. Liu, X. Wang, X. Wang, H. Han, C. Li, *J. Catal.* 2012, *293*, 61; d) G. Liu, J. Han, X. Zhou, L. Huang, F. Zhang, X. Wang, C. Ding, X. Zheng, H. Han, C. Li, *J. Catal.* 2013, *307*, 148.

[9] a) L. Rimoldi, A. Giordana, G. Cerrato, E. Falletta, D. Meroni, *Catal. Today* 2019, *328*, 210; b) J. Yang, X. Zhang, H. Liu, C. Wang, S. Liu, P. Sun, L. Wang, Y. Liu, *Catal. Today* 2013, *201*, 195; c) M. Picquart, S. Castro-Garcia, J. Livage, C. Julien, E. Haro-Poniatowski, *J. Sol-Gel Sci. Techn.* 2000, *18*, 199; d) K. K. Akurati, A. Vital, J.-P. Dellemann, K. Michalow, T. Graule, D. Ferri, A. Baiker, *Appl. Catal. B-Environ.* 2008, *79*, 53.

[10] J. Liqiang, Q. Yichun, W. Baiqi, L. Shudan, J. Baojiang, Y. Libin, F. Wei, F. Honggang, S. Jiazhong, *Solar Energy Materials and Solar Cells* 2006, *90*, 1773.

[11] a) S. E. Braslavsky, A. M. Braun, A. E. Cassano, A. V. Emeline, M. I. Litter, L. Palmisano, V. N. Parmon, N. Serpone, *Pure Appl. Chem.* 2011, *83*, 931; b) M. Qureshi, K. Takanabe, *Chem. Mater.* 2017, *29*, 158; c) M. Bellardita, M. Feilizadeh, R. Fiorenza, S. Scirè, L. Palmisano, V. Loddo, *Photoch. Photobio. Sci.* 2022, *21*, 2139.

[12] a) K. Kukli, M. Ritala, M. Leskelä, *Chem. Mater.* 2000, *12*, 1914; b) J. Joo, T. Yu, Y. W. Kim, H. M. Park, F. Wu, J. Z. Zhang, T. Hyeon, *J. Am. Chem. Soc.* 2003, *125*, 6553.

[13] a)K. Nishio, T. Sei, T. Tsuchiya, *J. Ceram. Soc. Jpn.* 1999, *107*, 199; b) K. Nishio, T. Tsuchiya, in *Handbook of Sol-Gel Science and Technology: Processing, Characterization and Applications*, (Eds: L. Klein, M. Aparicio, A. Jitianu), Springer International Publishing, Cham 2018.

[14] R. Deshmukh, M. Niederberger, *Chem. Eur. J.* 2017, *23*, 8542.

[15] a) M. Andrianainarivelo, R. Corriu, D. Leclercq, P. H. Mutin, A. Vioux, *J. Mater. Chem.* 1996, *6*, 1665; b) S. Acosta, R. Corriu, D. Leclercq, P. H. Mutin, A. Vioux, *J. Sol-Gel Sci. Techn.* 1994, *2*, 25; c) P. Arnal, R. J. P. Corriu, D. Leclercq, P. H. Mutin, A. Vioux, *Chem. Mater.* 1997, *9*, 694; d) H. Weingarten, J. R. Van Wazer, *J. Am. Chem. Soc.* 1965, *87*, 724.

[16] a) R. Scarfiello, C. T. Prontera, M. Pugliese, G. V. Bianco, G. Bruno, C. Nobile, S. Carallo, A. Fiore, T. Sibillano, C. Giannini, R. Giannuzzi, L. Carbone, G. Gigli, V. Maiorano, *Nanotechnology* 2021, *32*, 215709; b) A. Chemseddine, T. Moritz, *Eur. J. Inorg. Chem.* 1999, *1999*, 235.

[17] a)H. Jin, J. Zhou, J. Tao, Y. Gu, E. Kan, Z. Yao, B. Ouyang, *Carbon* 2024, *216*, 118571; b) Y. Fei, W. Jiao, Z. Wu, Z. Yang, W. Cheng, R. Che, *Nanoscale* 2023, *15*, 12193; c) M. Green, X. Chen, *Journal of Materiomics* 2019, *5*, 503.

[18] L. Liang, W. Gu, Y. Wu, B. Zhang, G. Wang, Y. Yang, G. Ji, *Adv. Mater.* 2022, *34*, 2106195.

[19] J. Liu, S. Yu, W. Zhu, X. Yan, *Appl. Catal. A-General* 2015, *500*, 30.
